# Oral Celastrol Nanomedicine Targeting Intestinal Antigen‐Presenting Cells to Effectively Mitigate Autoimmune Uveitis via Gut‐Retina Axis

**DOI:** 10.1002/advs.202519503

**Published:** 2026-02-03

**Authors:** Jinrun Chen, Yuqin Wu, Bofei Xu, Jiabei Hou, Yijing Li, Yuhan Hu, Yutuo Zhu, Wenqiao Zhang, Shuqi Feng, Huanting Jin, Yuchen Cheng, Yuanyuan Jin, Jianhong Zhou, Xingyi Li

**Affiliations:** ^1^ National Engineering Research Center of Ophthalmology and Optometry Eye Hospital Wenzhou Medical University Wenzhou China; ^2^ State Key Laboratory of Medicinal Chemical Biology Key Laboratory of Bioactive Materials Ministry of Education, and College of Life Sciences Nankai University Tianjin China

**Keywords:** APCs targeting, autoimmune uveitis, celastrol, nanomedicine, oral administration

## Abstract

Activation of retina‐specific CD4^+^ T cells capable of breaking through the blood‐retinal barrier (BRB) was significantly associated with the onset and progression of autoimmune uveitis (AU). Antigen‐presenting cells (APCs) orchestrate this process by presenting retinal antigens to naïve CD4^+^ T cells and driving their differentiation into autoreactive CD4^+^ T cells. Here, we report an intestinal APCs‐targeted strategy for treating AU based on orally administered nanoCEL (diameter: 37.06 ± 0.12 nm), a pH‐responsive nanomedicine exhibiting a great gastric acid stability and a pH‐responsive drug release behavior. After oral administration, nanoCEL effectively penetrates the intestinal mucus barrier and targets APCs in the intestine. Using an experimental autoimmune uveitis rat model, oral administration of nanoCEL (2 mg/kg) exhibits a superior therapeutic efficacy than free CEL treatment by suppressing the antigen‐presenting ability of APCs and impairing pathogenic T cell differentiation. Additionally, nanoCEL medication protects the BRB from the damage of pathogenic T cells by the reduction of the infiltration of peripheral immune cells and the activation of retinal glial cells. Compared to free CEL, oral administration of nanoCEL remarkably enhances drug bioavailability and improves biosafety without apparent systemic toxicity. Thus, the proposed APC‐targeted nanodrug delivery system might be a promising strategy to treat AU.

## Graphical abstract

Oral nanoCEL exhibits effective intestinal targeting of antigen‐presenting cells and restores the Th17/Treg balance in lymph nodes and spleen, ultimately protecting the blood‐retinal barrier by inhibiting peripheral immune cell infiltration and suppressing retinal glial cell activation.

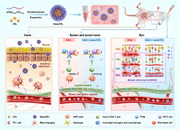

## 1 Introduction

Autoimmune uveitis is a severe sight‐threatening disease generally characterized by chronic and recurrent autoimmune intraocular inflammation, particularly affecting the uvea (i.e., iris, ciliary body, and choroid), retina, retinal vessels, and vitreous body [1, 2, 3]. Clinically, it often occurs with systemic autoimmune syndromes like spondylarthritis, Bechet's disease, and Vogt‐Koyanagi‐Harada disease (VKH). Among the well‐known pathogenesis of AU, the activated retinal antigen‐specific CD4^+^ T cells, primarily including regulatory (Treg) and effector T (i.e., Th1 and Th17) cells, are considered as the dominant drivers of AU [4, 5, 6]. Generally, corticosteroids alone or in combination with other immunosuppressive agents are the mainstay in clinical practice, but cause severe side effects and present limited efficacy [7, 8, 9]. Therefore, developing safe and effective treatment regimens for autoimmune uveitis is still an unmet medical requirement.

During the pathogenesis of autoimmune uveitis, dendritic cells (DCs) play a vital role in priming pathogenic T cells and generating the inflammatory milieu, thereby being regarded as a logical target to modulate immunity [10, 11, 12, 13, 14]. Inducing immune tolerance with DCs‐targeted nano‐formulation is a promising immunotherapy strategy for various autoimmune disorders. Encouraged by the initial success in DCs‐targeted nanomedicine for COVID‐19 infections [15, 16], this immunotherapy strategy is starting to be adapted and flourishing. Similar to invaded pathogens, nanoparticles administered into the circulatory or lymphatic systems are primarily captured by antigen‐presenting cells (APCs), including macrophages, DCs, and so on [17, 18, 19]. Several physicochemical parameters of nanoparticles, including the bulk materials forming the nanoparticles, size, shape, surface, and charge, significantly influence the APCs targeting and modulation [20, 21, 22]. Nanoparticles with a diameter less than 100 nm can be effectively taken up by DCs via clathrin‐mediated endocytosis and reach lymph‐node‐resident DCs within hours of systemic injection [23, 24, 25]. Apart from the size and shape of nanoparticles, the surface chemistry of nanoparticles (i.e., hydrophilicity and charge) also remarkably affects their interaction and internalization into DCs [26, 27, 28]. Numerous studies have shown that negatively charged nanoparticles are more prone to reduce acute inflammation in immune disorders, and the PEGylation of nanoparticles with shorter PEG chains (2 and 3 kDa) displays higher DCs targeting than that of polyvinyl alcohol (PVA) modification [28, 29, 30]. Thus, the optimized properties of DCs‐targeted nanoparticles might be a powerful strategy to deliver therapeutic agents for treating autoimmune uveitis.

Celastrol (CEL) is one of the valuable natural bioactive compounds derived from Tripterygium wilfordii Hook F (a traditional Chinese herb known as thunder god vine) [31]. Over the past few years, a set of studies have elucidated that CEL inhibits tumor growth, suppresses metastasis and angiogenesis [32, 33, 34], and displays a potent immunological suppression ability in the management of rheumatoid arthritis, autoimmune uveitis, psoriasis, and multiple sclerosis [35, 36, 37, 38]. However, the clinical application of CEL is confronted with severe constraints owing to its poor water solubility (11 µg/mL at pH of 7.4), low bioavailability, and high off‐target effects, which result in severe organ toxicity, notably hepatotoxicity, nephrotoxicity, and cardiotoxicity [35, 39, 40]. To realize better clinical application of CEL with specific cell targeting and enhanced biosafety, numerous strategies have been implemented, mainly including the development of its derivatives and nanodrug delivery systems (NDDSs). Several types of NDDSs, such as liposomes, polymeric micelles, lipid nanoparticles, and exosomes, have been developed to encapsulate CEL to provide better solubility and bioavailability, enhanced therapeutic efficacy, and lower systemic toxicity [41, 42, 43]. Inspired by previous studies, we here formulated a nanodrug delivery system based on polymeric micellization (i.e., methoxy poly (ethylene glycol)‐block‐poly (ε‐caprolactone); MPEG‐PCL), namely nanoCEL, for APCs targeting and modulation to treat autoimmune uveitis. The proposed nanoCEL was expected to efficiently uptake by mesenteric lymph nodes (MLN) after oral administration owing to its small particle size and robust mucosal permeability, and subsequently orchestrate the T‐cells differentiation to protect the integrity of BRB via a gut‐retina axis. Hopefully, this oral APCs‐targeted nanoCEL can offer a promising avenue for advancing autoimmune uveitis treatment strategies.

## 2 Materials and Methods

### 2.1 Materials

Methoxy poly (ethylene glycol)‐block‐poly (ε‐caprolactone) polymer (MPEG‐PCL; M_w_ = MPEG2000/PCL3000) was synthesized by our group. Celastrol (CEL) and Nile red (NR) were provided by Aladdin Reagent Co., Ltd (Shanghai, China). Simulated gastric fluid (SGF) and simulated intestinal fluid (SIF) were purchased from Shanghai Yuanye Bio‐Technology Co., Ltd (Shanghai, China). IRBP R16 was obtained from Shanghai Sangon Biological Engineering Technology & Services Co., Ltd (Shanghai, China). *Mycobacterium tuberculosis* H37Ra was procured from Difco Laboratories (Detroit, MI, USA). All other reagents used in this study were of analytical grade.

### 2.2 Fabrication of CEL‐loaded MPEG‐PCL Micelles (nanoCEL)

MPEG‐PCL block polymer was first synthesized according to the previous works [44, 45, 46]. Then, nanoCEL was fabricated by a facile thin‐film hydration method. Briefly, a certain amount of CEL and MPEG‐PCL block polymer was co‐dissolved into acetone (10 mL), and then the solvent was evaporated using a rotary evaporator (EYELA, N‐1300SWB). After that, distilled water (10 mL) was added, and the resulting residue was re‐suspended into a clear solution at 55℃ to afford nanoCEL. Finally, the obtained nanoCEL was filtered by a 0.22‐µm Millipore filter and lyophilized into powder for further usage.

### 2.3 Characterization of nanoCEL

Drug loading capacity (LC) and encapsulation efficiency (EE) of nanoCEL were measured using a high‐performance liquid chromatography (HPLC) assay. An indicated amount of nanoCEL powder was re‐suspended in a distilled water solution at 55℃ to give a transparent nanoCEL solution. The drug content was thereafter quantified by an HPLC (Agilent 1200, Agilent Technologies, USA). HPLC analysis was conducted on a reverse‐phase C18 column (4.6 × 150 mm, 5 µm; Agilent Technologies) at 30℃. The mobile phase was composed of methanol and glacial acetic acid (90:10; v/v), and the eluent was detected at a wavelength of 425 nm. LC and EE of nanoCEL were calculated as follows:

(1) $$\mathrm{LC}\;\left(\%\right)=\frac{\mathrm{drug}\;\mathrm{in}\;\mathrm{nanoCEL}}{\mathrm{total}\;\mathrm{amount}\;\mathrm{of}\;\mathrm{nanoCEL}}\times 100\;$$

(2) $$\mathrm{EE}\;\left(\%\right)=\frac{\mathrm{drug}\;\mathrm{in}\;\mathrm{nanoCEL}}{\mathrm{the}\;\mathrm{total}\;\mathrm{drug}}\;\times 100\;$$

The size distribution and zeta potential of nanoCEL were measured by a Zetasizer Nano ZS‐90 (Malvern Instruments, Malvern, UK). Samples were diluted with distilled water and measured at 25 ℃. Morphological observation was performed by transmission electron microscopy (Talos F200S, Thermo Fisher, USA). Samples were pipetted on a copper grid and negatively stained with 0.5% phosphotungstic acid before observation.

Fourier‐transform infrared (FTIR) analysis was used by an FTIR spectrometer (Tensor II, Bruker, Germany) within the range of 400–4000 cm^−1^. X‐ray diffraction (XRD) spectra of various samples were measured by an X‐ray diffractometer (D8 Advance, Bruker, Karlsruhe, Germany). Data were collected at scattering angles (2θ) ranging from 10 to 50°. Differential scanning calorimetry (DSC) profiles of different samples were detected by a DSC instrument (DSC 8000, Perkin‐Elmer Instruments, USA). Samples were sealed into an aluminum pan, and the DCS profile was recorded from 20 to 200℃.

### 2.4 In Vitro Drug Release and Stability Test

In vitro drug release of nanoCEL at different pH conditions (pH = 1.5, 4.5, and 7.5) was measured using a dialysis method. Briefly, nanoCEL (0.5 mL) was sealed into a dialysis bag (M_w_ = 8000–14,000 Da) and then immersed in release medium (10 mL) at 37℃ with continuous stirring (120 rpm) for a periodical study. The release medium was phosphate‐buffered saline (PBS, 0.1 mm) containing 0.1% Tween‐80, and its pH was adjusted to 1.5 and 4.5 using hydrochloric acid. At predetermined time points, 1 mL of release medium was collected, and the released CEL was quantified by an HPLC method described in Section 2.3. At each time interval, 1 mL of freshly prepared release medium was supplemented for the continuous study. Additionally, to simulate gastrointestinal conditions, nanoCEL was incubated with simulated gastric fluid (SGF) and simulated intestinal fluid (SIF), and the variance of drug content as a function of time was measured by a HPLC method as described in Section 2.3.

### 2.5 In Vitro Cellular Uptake Assay

To visualize the cellular uptake of nanomedicine in murine RAW264.7 macrophage (CL‐0190, Pricella, Wuhan, China), murine JAWSII dendritic cells (C2227, WHELAB, Shanghai, China), and Buffalo Rat Liver‐3A hepatocyte (BRL‐3A, CL‐0036, Pricella, Wuhan, China), NR‐tagged micelles named nanoNR were used. Briefly, RAW264.7 macrophage, JAWSII dendritic cells, or BRL‐3A hepatocytes were seeded in a 24‐well plate at a density of 5×10^5^ cells/well and then incubated overnight. After being challenged by lipopolysaccharide (LPS, 1 µg/mL) for 24 h, the culture medium was discarded, and the culture medium containing 10 µg/mL of free NR or nanoNR was added for 15 min of incubation. The cellular uptake was visualized by an inverted fluorescent microscope (Zeiss ELYRA 7 Confocal Laser Microscope, Zeiss, Jena, Germany) and analyzed by flow cytometry (BD Accuri C6 Plus, BD Biosciences, USA). Cells without any manipulation were set as the blank group.

To assess the endocytic pathways of LPS‐activated RAW264.7 macrophage, various endocytic inhibitors, including chlorpromazine (CPZ), 5‐(N, N‐dimethyl) amiloride (DMA), genistein (GEN), and sucrose (SUC), were employed. RAW264.7 macrophages were seeded in a 24‐well plate at a density of 5×10^5^ cells/well and then incubated overnight. After being stimulated by LPS (1 µg/mL) overnight, different kinds of inhibitors, including CPZ (10 µg/mL), DMA (10 µM), GEN (50 µg/mL), and SUC (154 mg/mL), were added for 30 min of incubation. Subsequently, the culture medium was replaced with a fresh culture medium containing nanoNR (10 µg/mL) for another 15 min of incubation. Finally, the cellular uptake of nanoNR was analyzed by flow cytometry (BD Accuri C6 Plus, BD Biosciences, USA).

### 2.6 In Vitro Anti‐inflammatory Efficacy and Inhibition of Antigen‐presenting Ability

Briefly, RAW264.7 macrophages or JAWSII dendritic cells were seeded in a 24‐well plate at a density of 1×10^5^ cells/well and cultured overnight. Following the treatment with 200 nm of free CEL or nanoCEL for 2 h, cells were stimulated with LPS (1 µg/mL) overnight. Thereafter, cell culture medium from each treatment was collected, and the levels of nitric oxide, IL‐6 (SM6000B, R&D, USA), TNF‐α (SMTA00B, R&D, USA), IL‐12p70 (SM1270, R&D, USA) and CXCL5 (MX000, R&D, USA) in supernatant were measured by Griess assay and ELISA kits, respectively. In addition, JAWSII dendritic cells were seeded in a 24‐well plate equipped with cell‐climbing slices at a density of 1×10^5^ cells/well and cultured overnight. After the pretreatment with 200 nm of free CEL or nanoCEL for 2 h, cells were challenged with LPS (1 µg/mL) overnight. The cell‐climbing slices were harvested and fixed with 4% paraformaldehyde for 20 min at room temperature, washed with PBS three times. After the permeabilization with 0.1% Triton X‐100 for 15 min, the samples were blocked with goat serum for 2 h. Thereafter, the samples were incubated with the anti‐CD11c (PA5‐90208, Invitrogen, 1:200) and anti‐MHC class II (14‐5321‐82, Invitrogen, 1:200) overnight, followed by the incubation of fluorescence‐conjugated secondary antibodies (Invitrogen, 1:500) at room temperature. Finally, the samples were stained with 4′,6‐diamidino‐2‐phenylindole (DAPI, Beyotime Biotechnology, China) and visualized by a confocal laser scanning microscope (CLSM, Zeiss LSM880, Zeiss, Jena, Germany). Image J software was used to analyze the mean fluorescence intensity.

### 2.7 In Vivo Mucus Permeability and Distribution in Intestinal Tissue

To visualize the mucus permeability and distribution of nanomedicine in intestinal tissue after oral administration, NR‐tagged micelles (nanoNR) were used. At day 14 post‐immunization, EAU rats were anesthetized, and a 4 cm length of intestine was ligated at both ends. After that, 1 mL of either nanoNR or free NR (0.1 mg/mL) was gently injected into the intestinal lumen [47]. One hour later, the rats were sacrificed, and the ligated tissues were excised and then washed with PBS in triplicate. Finally, the ligated intestinal tissues were incubated with Alexa Fluor 488‐conjugated wheat germ agglutinin (W11261, Invitrogen, 1:100) to visualize the mucus permeability. Additionally, the ligated intestinal tissues were cryo‐sectioned (∼10 µm of thickness) and incubated with primary antibodies of CD4 (14‐0041‐82, Invitrogen, 1:100) and CD11b/c (ab1211, Abcam, 1:200) at 4℃ overnight. Following the incubation with secondary antibodies, the distribution of nanoNR was visualized using a CLSM (Zeiss LSM880, Zeiss, Jena, Germany).

### 2.8 In Vitro Intestinal Epithelial Barrier Penetration and Cellular Uptake

According to previous studies [48, 49], a 3D trans‐well coculturing model using Caco‐2 cells (CL‐0050, Pricella, Wuhan, China) was established to assess in vitro intestinal epithelial barrier penetration. Briefly, Caco‐2 cells were seeded into MILLICELL‐PCF 12‐well culture plate at a density of 5×10^4^ cells/well and cultured for 21 days until the transepithelial electrical resistance (TEER) approached a constant value of ∼500 Ω/cm^2^. After washing with Hank's balanced salt solution three times, the rinsed Caco‐2 monolayer cells at the upper chamber were transferred into a 12‐well plate with the RAW264.7 macrophages or JAWSII dendritic cells at a density of 5×10^5^ cells/well in the lower chamber. Thereafter, 0.5 mL of either free NR or nanoNR was added to the upper chamber to give a final concentration of NR at 40 µg/mL. After 2 h of co‐incubation, cells from the lower chamber were harvested, and the cellular uptake of NR was visualized and analyzed by a CLSM (Zeiss LSM880, Zeiss, Jena, Germany) and flow cytometry (BD Accuri C6 Plus, BD Biosciences, USA), respectively.

### 2.9 Induction of Experimental Autoimmune Uveitis (EAU) and Assessment of Therapeutic Efficacy

All animal experiments comply with the Guide for the Care and Use of Laboratory Animals, Institute of Laboratory Animal Resources. They were approved by the Institutional Animal Care and Use Committee of Wenzhou Medical University (YSG23103019). As reported previously [50], EAU was induced in Lewis rats by the subcutaneous injection of 0.1 mL of emulsion with complete Freund's adjuvant containing IRBP R16 and *Mycobacterium tuberculosis* H37Ra at the left hind footpads. After immunization, the rats were medicated with PBS, blank micelles, 2 mg/kg free CEL, and 1 or 2 mg/kg nanoCEL via an oral administration route. At specific time intervals, the clinical signs of EAU were observed and graded using a slit‐lamp biomicroscope (SLM‐8E, Chongqing Kang Hua Rui Ming Technology Co., LTD, China) by an experienced ophthalmologist based on the established criteria [51].

At day 11 post‐immunization, optical coherence tomography (OCT) was employed to evaluate the impact of various formulations on the retinal architecture and vitreous humor using a Micron IV (Phoenix Research Laboratories, USA). Additionally, the visual function of rats from each group was analyzed by a specialized Ganzfeld dome system (Roland Q400, Wiesbaden, Germany), and the scotopic ERG responses at a light intensity of 3 cd.s/m^2^ were recorded.

At day 14 post‐immunization, rats from each group were sacrificed, and the eyeballs were harvested. After fixation in FAS eyeball fixative solution (G1109, Servicebio, China) for 24 h at room temperature, the specimens were dehydrated with graded ethanol solution and embedded in paraffin. Thereafter, the paraffins were sectioned (∼5 µm of thickness) and stained with hematoxylin and eosin (H&E, Beyotime Biotechnology, China) for histopathological observation. According to the previously established standard [52], the histopathological severity of EAU from each group was scored. Additionally, the intestinal tissue from each group was excised. The harvested eyeball and intestine were embedded in optimal cutting temperature compound and cryo‐sectioned (∼10 µm of thickness). The sections were blocked with blocking buffer (P0102, Beyotime Biotechnology, China) for 2 h, followed by incubating primary antibodies at 4℃ overnight. The used primary antibodies were as follows: Iba1 (019‐19741, Wako, 1:400), GFAP (ab7260, Abcam, 1:400), CD4 (14‐0041‐82, Invitrogen, 1:100), IL‐17 (SC‐374218, Santa Cruz, 1:50), CD11b/c (ab1211, Abcam, 1:200), and MHC class II (14‐0920‐82, Invitrogen, 1:200). After incubation with the secondary antibodies, the sections were stained with DAPI (Beyotime Biotechnology, China) and visualized by a CLSM (Zeiss LSM880, Zeiss, Jena, Germany). Image J software was used to analyze the mean fluorescence intensity.

### 2.10 Activation of APCs and T Cell Differentiation in EAU Rat

At day 14 post‐immunization, the rats from each group were sacrificed. The spleen, cervical lymph nodes (CLN), mesenteric lymph nodes (MLN), and retina were excised. The harvested spleen was grinded and lysed with 1 mL of RBC lysis buffer (C3702, Beyotime Biotechnology, China). The excised CLN, MLN, and retina were grinded and homogenized to afford a suspension. The lysate or homogenate was mixed with 3 mL of cell staining buffer (420201, Biolegend, USA) and then centrifuged at 5000 rpm for 5 min. The resulting residue was resuspended in 1 mL of cell staining buffer and filtered through a 300‐mesh cell sieve to generate a single‐cell suspension. The obtained cell suspension was incubated with Alexa Fluor 700‐CD45 (202218, Biolegend, 1:200), PerCP‐Cy5.5‐CD11b/c (201819, Biolegend, 1:200), Elab Fluor Red 780‐CD3 (E‐AB‐F1228US, Elabscience, 1:200), FITC‐CD4 (E‐AB‐F1105UC, Elabscience, 1:200), PE/Cyanine7‐CD8 (E‐AB‐F1098H, Elabscience, 1:200), BV421‐CD25 (565608, BD Bioscience, 1:200), eFluor660‐IFN‐γ (50‐7310‐80, Invitrogen, 1:200), BV786‐IL‐17A (417‐7177‐82, Invitrogen, 1:80), and eFluor506‐Foxp3 (69‐5773‐82, Invitrogen, 1:40) antibodies at 4°C for 30 min, and then analyzed by a flow cytometer (Attune NxT, Invitrogen, USA).

### 2.11 The Assessment of Blood‐Retina Barrier (BRB) Integrity

The BRB integrity of EAU rats from each group at day 14 post‐immunization was examined by Evans blue perfusion assay and zonula occludens‐1 (ZO‐1) immunofluorescence assay, respectively. For the Evans blue perfusion assay, EAU rats from each group were intraperitoneally injected with 1 mL of Evans blue (2%) aqueous solution and sacrificed at 2 h post‐injection. The excised retinas from each group were flat‐mounted and observed by a CLSM (Zeiss LSM880, Zeiss, Jena, Germany). With regard to ZO‐1 immunofluorescence assay, the excised retinal pigment epithelium‐choroid‐sclera complex was flat‐mounted and then incubated with a blocking buffer for 2 h at room temperature. Thereafter, the mounted tissue was immersed in primary antibodies of ZO‐1 (61‐7300, Invitrogen, 1:400) and CD45 (550566, BD Bioscience, 1:200) at 4℃ overnight, and then incubated with the secondary antibodies, followed by the visualization using a CLSM (Zeiss LSM880, Zeiss, Jena, Germany).

### 2.12 Pharmacokinetics and Biodistribution of nanoCEL After Oral Administration

To measure the plasma pharmacokinetics, the rats were orally administered with either 2 mg/kg of nanoCEL or free CEL. At predetermined time points, the whole blood sample was collected via the tail vein and then centrifuged at 2500 rpm for 10 min to obtain the plasma. Additionally, the rats at 6 h post medication were sacrificed, and the major organs, including heart, liver, spleen, lung, and kidney, were harvested to investigate drug biodistribution. The homogenized organ tissues and plasma were extracted with ethyl acetate. Then the drug contents were quantified using an ultra‐performance liquid chromatography triple quadrupole mass spectrometer (UPLC‐MS/MS, QTrap 6500+, AB Sciex, USA). UPLC analysis was conducted on an ACQUITY UPLC BEH C18 column (2.1 × 100 mm, 1.7 µm; Waters) at 40℃. In negative ESI modes, the mobile phase of A = 1% formic acid aqueous solution and B = acetonitrile. The gradient started at 10% B for 2 min, followed by a linear increase to 95% B over 0.2 min, was maintained for 4 min, and then reduced to 5% in 2 min. The eluent was detected at a wavelength of 425 nm. The ESI source conditions were configured as follows: ion source Gas1 and ion source Gas2, 50 Psi; curtain gas, 30 Psi; source temperature, 450 C; and Ion Spray Voltage Floating (ISVF), −4500 V.

### 2.13 Biosafety Assessment

To assess the systemic toxicity of CEL, the rats were medicated daily with PBS, blank micelles, 2 mg/kg of free CEL, or nanoCEL via an oral administration route for a continuous 14 days. At day 14, the rats from each group were sacrificed, and the whole blood was withdrawn for the quantification of aspartate aminotransferase/alanine transaminase (AST/ALT), gamma‐glutamyl transferase (GGT), Alb/Globin (A/G), Urea, and Uric Acid (UA) using a BS‐800M automatic biochemical analyzer (Mindray, Shenzhen, China). Additionally, the major organs, including the heart, liver, spleen, lung, and kidney, were harvested for histopathological observation using an H&E staining. To calculate the median lethal dose (LD_50_) value of CEL, the rats were orally administered different doses of free CEL and nanoCEL once, respectively. The toxic symptoms, number of deaths, and time of death in each group of rats were recorded within 24 h after oral administration. The LD_50_ value and 95% confidence interval were calculated using the Bliss method.

### 2.14 Statistical Analysis

All data collected in this study are expressed as mean ± standard error of the mean (SEM). Clinical scores were compared using two‐way ANOVA for repeated measures, followed by post hoc Bonferroni's *t*est. One‐way ANOVA was conducted to assess differences between groups (in cases where there were more than three groups). An unpaired Student's *t*‐test was utilized to analyze differences between the two groups. Significance levels were denoted as ^*^ *p* < 0.05, ^**^ *p* < 0.01, or ^***^ *p* < 0.001.

## 3 Results and Discussion

### 3.1 Synthesis and Characterization of nanoCEL

**FIGURE 1:**
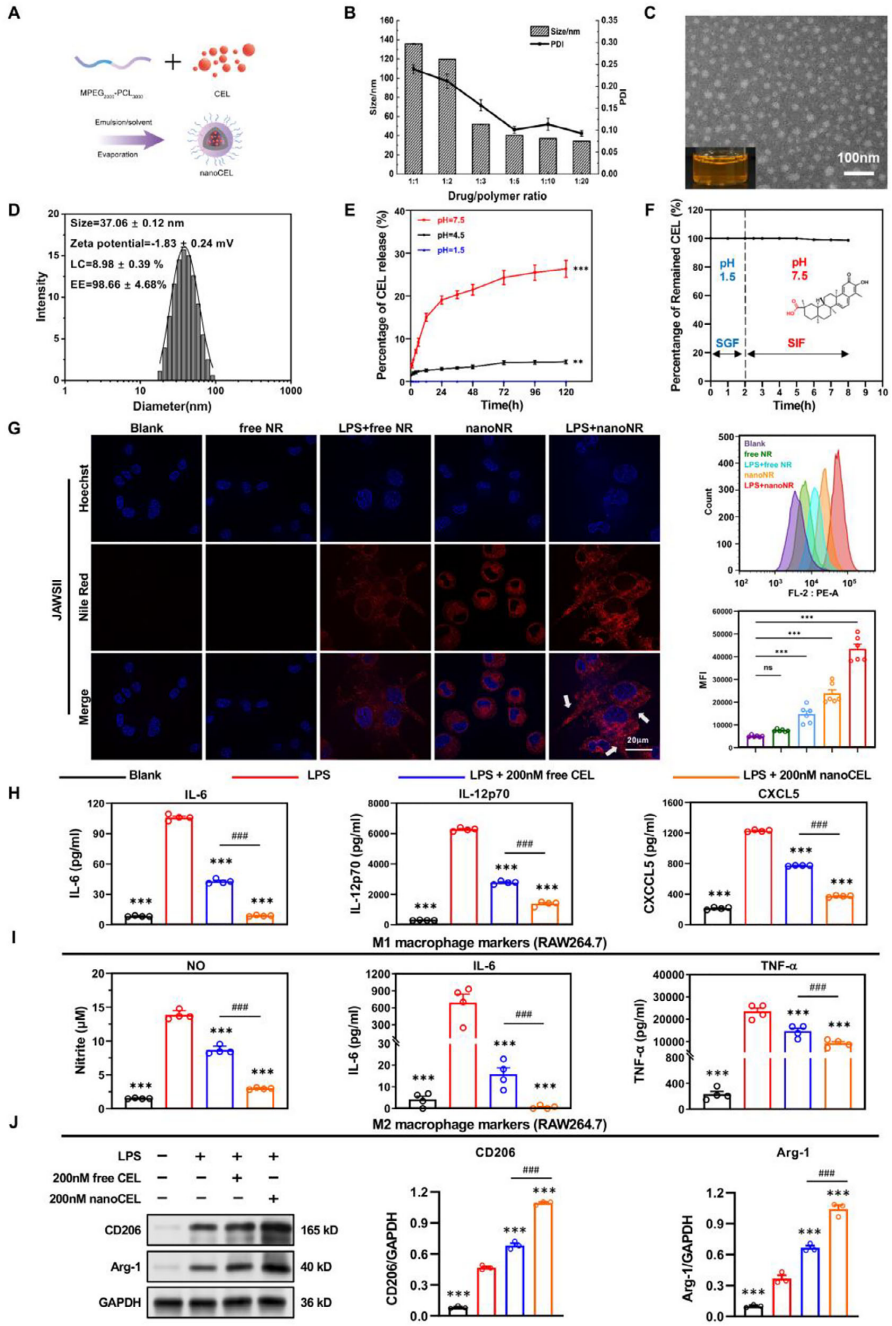
Preparation of nanoCEL and its immune‐modulation properties in vitro. (A) Schematic fabrication of nanoCEL; (B) Effect of drug/polymer feed ratio on the mean particle size and polydispersity index (PDI) of nanoCEL; (C) The optical (inset) and TEM images of nanoCEL; (D) Particle size distribution, zeta potential, loading capacity (LC), and encapsulation efficiency (EE) of nanoCEL; (E) In vitro release profiles of nanoCEL under varied pH conditions (PBS, pH = 1.5, 4.5, and 7.5) at 37°C for 120 h (n = 3; ^**^ *p* < 0.01, ^***^ *p* < 0.001 vs. pH = 1.5); (F) In vitro stability of nanoCEL in simulated gastric fluid (SGF) at 37°C for 2 h, followed by simulated intestinal fluid (SIF) at 37°C for 6 h (n = 3); (G) CLMS images and flow cytometry analysis of JAWSII dendritic cell uptake of various formulations after incubation for 15 min. Nile Red (NR) fluorescence is depicted in red. (n = 6; ns indicates no significance, ^***^ *p* < 0.001 vs. blank group); (H) IL‐6, IL‐12p70, and CXCL5 levels in the cell culture medium of LPS‐stimulated JAWSII dendritic cells after treatment with 200 nm free CEL and 200 nm nanoCEL; cells without any manipulation set as blank group (n = 4; ^***^ *p* < 0.001 vs. LPS‐stimulated group; ^###^ *p* < 0.001). (I) NO, IL‐6, and TNF‐α levels in the cell culture medium of LPS‐stimulated RAW264.7 macrophages after treatment with 200 nm free CEL and 200 nm nanoCEL; cells without any manipulation set as blank group (n = 4; ^***^ *p* < 0.001 vs. LPS‐stimulated group; ^###^ *p* < 0.001). (J) Western blot analysis of CD206 and Arg‐1 levels from each group (n = 3; ^***^ *p* < 0.001 vs. LPS‐stimulated group; ^###^ *p* < 0.001).

Here, we reported a nanodrug delivery system that targeted APCs to effectively deliver CEL into the cytosol and to modulate the antigen‐presentation function of APCs and differentiation of pathological Th17 cells and Th1 cells, thus attenuating autoimmune uveitis. As reported previously [44], nanoCEL was fabricated by a facile thin‐film hydration method (Figure 1A). By modulating the drug‐polymer ratios, a set of nanoCEL formulations were obtained (Table S1). With decreasing drug‐polymer ratios, the particle size as well as polydispersity (PDI) of nanoCEL decreased accordingly (Figure 1B). The optimized nanoCEL at a drug/polymer feed ratio of 1:10 was used for further application. TEM observation of nanoCEL showed the spherical morphology with mean particle diameter of 37.06 ± 0.12 nm. Zeta potential of nanoCEL was slightly negatively charged (−1.83 ± 0.24 mV). LC and EE of nanoCEL were 8.98 ± 0.39% and 98.66 ± 4.68%, respectively (Figure 1C,D). FTIR analysis indicated that CEL was physically entrapped into MPEG‐PCL micelles to afford nanoCEL with the existence of the characteristic absorption peak of both CEL and MPEG‐PCL micelles (Figure S1A). Notably, CEL amorphously existed in nanoCEL, as indicated by XRD and DSC measurements (Figure S1B,C).

### 3.2 NanoCEL Exhibits a pH‐sensitive Drug Release Behavior

To explore the drug release performance of nanoCEL after oral administration, an in vitro drug release study of nanoCEL was conducted at different pH conditions (pH = 1.5, 4.5, and 7.5). As shown in Figure 1E, nanoCEL exhibited distinct release behaviors in different pH conditions. The drug was almost not released from nanoCEL at pH of 1.5, while about 5% of the total drug was released from nanoCEL at pH of 4.5 during the whole study period. Notably, the drug release rate of nanoCEL elevated significantly at pH of 7.5, reaching a cumulative release of approximately 30% after 120 h. All these results indicated that nanoCEL exhibited a pH‐responsive drug release behavior. This exceptional pH‐responsive drug release might be ascribed to the ionization of CEL at weak alkali conditions (pH = 7.5) to provide rapid drug release behavior, but not at acidic conditions (pH = 1.5 and 4.5). To further assess the stability of nanoCEL in the gastrointestinal tract, nanoCEL was incubated with the simulated gastric fluid (SGF, pH = 1.5) with trypsin and simulated gastrointestinal fluid (SIF, pH = 7.5) at 37℃. Drug content of nanoCEL did not decay in both SGF and SIF medium, suggesting that the nanoCEL displayed great drug stability in the gastrointestinal environment (Figure 1F).

### 3.3 NanoCEL Enhances In Vitro Cellular Uptake, Anti‐inflammation, and Suppression of Antigen‐presenting Ability

Since APCs, including macrophages, dendritic cells, and so on, have been elucidated to be vital roles in the onset and progression of autoimmune disorders [53], we thereafter adopted RAW264.7 macrophages and JAWSII dendritic cells to assess the in vitro performance of nanoCEL. To visualize the cellular uptake of micelles, NR‐tagged micelles (nanoNR) were used. As shown in Figure 1G, nanoNR enhanced the cellular uptake in JAWSII dendritic cells with respect to free NR, corresponding to the higher intensity of red fluorescence. It is worth noting that the cellular uptake of nanoNR was significantly elevated in LPS‐stimulated JAWSII dendritic cells as compared to that in JAWSII dendritic cells. Flow cytometry analysis also identified that LPS‐stimulated JAWSII dendritic cells exhibited the highest cellular uptake of nanoNR among all tested samples. All these results implied that micellization of NR remarkably enhanced the cellular uptake of NR in both of JAWSII dendritic cells and LPS‐stimulated JAWSII dendritic cells. Similarly, nanoNR exhibited the highest cellular uptake in LPS‐stimulated RAW264.7 macrophages (Figure S1D). We thereafter assessed the endocytic pathway of nanoNR in LPS‐stimulated RAW264.7 macrophages. Following by the treatment with chlorpromazine (CPZ) and sucrose (SUC), the cellular uptake of nanoNR was remarkably suppressed, suggesting that clathrin and scavenger receptor‐dependent endocytic pathways dominantly mediated the endocytosis of nanoNR (Figure S1E).

**FIGURE 2:**
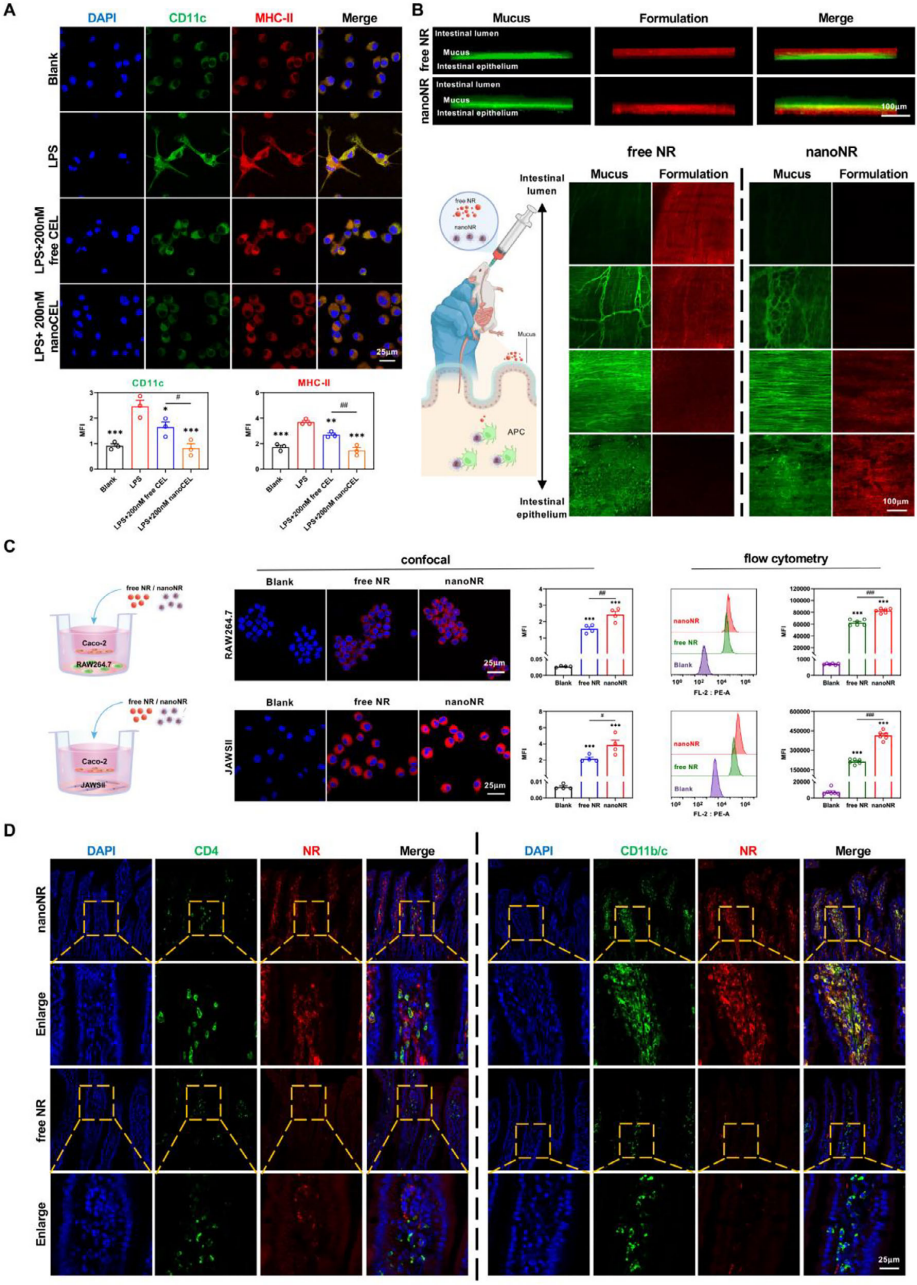
The intestinal mucus penetration and APCs‐targeting performances of nanoCEL after oral administration. (A) Co‐immunofluorescence staining of CD11c (green) and MHC‐II (red) within JAWSII dendritic cells after treatments with the indicated formulations. (n = 3; ^*^ *p* < 0.05, ^**^ *p* < 0.01, ^***^ *p* < 0.001 *vs*. LPS‐stimulated group; ^#^ *p* < 0.05, ^##^ *p* < 0.01); (B) Representative 3D and 2D CLSM images of intestinal mucus layer penetration of free NR and nanoNR. Nile Red (NR) fluorescence is depicted in red; (C) Representative CLMS images and flow cytometry analysis of RAW264.7 macrophages and JAWSII dendritic cells from each group. (n = 4 for CLMS analysis and n = 6 for flow cytometry analysis; ^***^ *p* < 0.001 vs. blank; ^#^ *p* < 0.05; ^##^ *p* < 0.01; ^###^ *p* < 0.001); (D) Representative CLMS images of NR distribution in intestinal tissues after treatments with nanoNR and free NR. The CD4^+^ cells and CD11b/ccells were represented as green.

Taking into consideration of the enhanced cellular uptake of nanoNR with respect of free NR, we further investigated the in vitro pharmacological activities of nanoCEL in RAW264.7 macrophages and JAWSII dendritic cells. As shown in Figure 2A, the stimulation of LPS resulted in the high expression of CD11c and MHC‐II, as well as cell deformation, which were the indicators of antigen‐presenting ability, in JAWSII dendritic cells. Both of free CEL and nanoCEL greatly suppressed the expression of CD11c and MHC‐II in LPS‐stimulated JAWSII dendritic cells, and the treatment of nanoCEL led to the more profound inhibitory effect as compared to the treatment of free CEL. Additionally, nanoCEL remarkably suppressed the production of IL6, IL‐12p70, and CXCCL5 in LPS‐stimulated JAWSII dendritic cells (Figure 1H). These results implied that the nanoCEL displayed the more potent inhibitory effect of antigen‐presenting ability than free CEL. Similarly, the stimulation of LPS caused the RAW264.7 macrophages polarization into M1 and M2 phenotypes, corresponding to the high expression of nitrite, IL‐6, TNF‐α, and upregulation of CD206 and Arg‐1 (Figure 1I,J). The treatment of free CEL significantly inhibited the production of nitrite, IL‐6, and TNF‐α, and upregulated the level of CD206 and Arg‐1 in LPS‐stimulated RAW264.7 macrophages. Notably, the treatment of nanoCEL exhibited the more potent inhibitory effect of these cytokines (i.e., nitrite, IL‐6, TNF‐α) and upregulation of CD206 and Arg‐1 in LPS‐stimulated RAW264.7 macrophages. These results strongly indicated that the proposed nanoCEL possessed robust capacity to reverse the macrophage phenotype from M1‐like type to M2‐like type, thus possessing the potent anti‐inflammatory efficacy.

### 3.4 NanoCEL Enhances Mucus Penetration and Targets APCs in Intestine After Oral Administration

To assess the ability of nanoCEL to penetrate the mucus layer after oral administration, the fluorescence‐tagged micelles (nanoNR) were used for measurements in ex vivo intestine. CLSM images showed that free NR was primarily located on the surface of the mucus layer at 1 h of incubation. Conversely, nanoNR resulted in deeper location of the mucus layer (Figure 2B). Apparently, micellization of NR using MPEG‐PCL polymer enhanced intestinal mucus permeability. This result might be explained by the fact that PEGylation of micelles rapidly diffused across mucus owing to the reduced interaction between micelles and mucus in the presence of hydrophilic PEG‐shell. It has also been documented that PEGylation of nanoparticles increased the random Brownian movement, thus facilitating mucus penetration [54]. Thereafter, Caco‐2 cells were cultured as a monolayer in a trans‐well model to mimic the intestinal epithelial barrier (IEB) at the upper chamber, and the RAW264.7 macrophages and JAWSII dendritic cells were cultured in the lower chamber. Both of free NR and nanoNR efficiently penetrated IEB to approach the lower chamber, and were then uptaken by RAW264.7 macrophages and JAWSII dendritic cells (Figure 2C). Markedly, the higher uptake efficacy in RAW264.7 macrophages and JAWSII dendritic cells was achieved by nanoNR compared with free NR, indicating a robust barrier permeability of micellization. Encouraged by the enhanced penetration in the mucus layer and IEB, we further assessed the in vivo performance of nanoNR in rats. As presented in Figure 2D, either free NR or nanoNR (red fluorescence signal) did not co‐localize with CD4^+^ T cells (green fluorescence signal). Notably, an apparent red fluorescence signal of nanoNR was intensively co‐localized with CD11b/c^+^ APCs cells (green fluorescence signal) but not in the free NR group. This result indicated that nanoNR rather than free NR could be selectively phagocytosed by CD11b/c^+^ APCs cells but not by CD4^+^ T cells. It has been reported that APCs, including macrophages and dendritic cells, may serve as powerful cellular vehicles to hitchhike nanoparticles [20, 55]. All these data demonstrated that nanoNR promoted the intestinal mucus permeability, accompanied by APCs targeting in the intestine after oral administration.

### 3.5 NanoCEL Overwhelmingly Ameliorates Autoimmune Intraocular Inflammation

**FIGURE 3:**
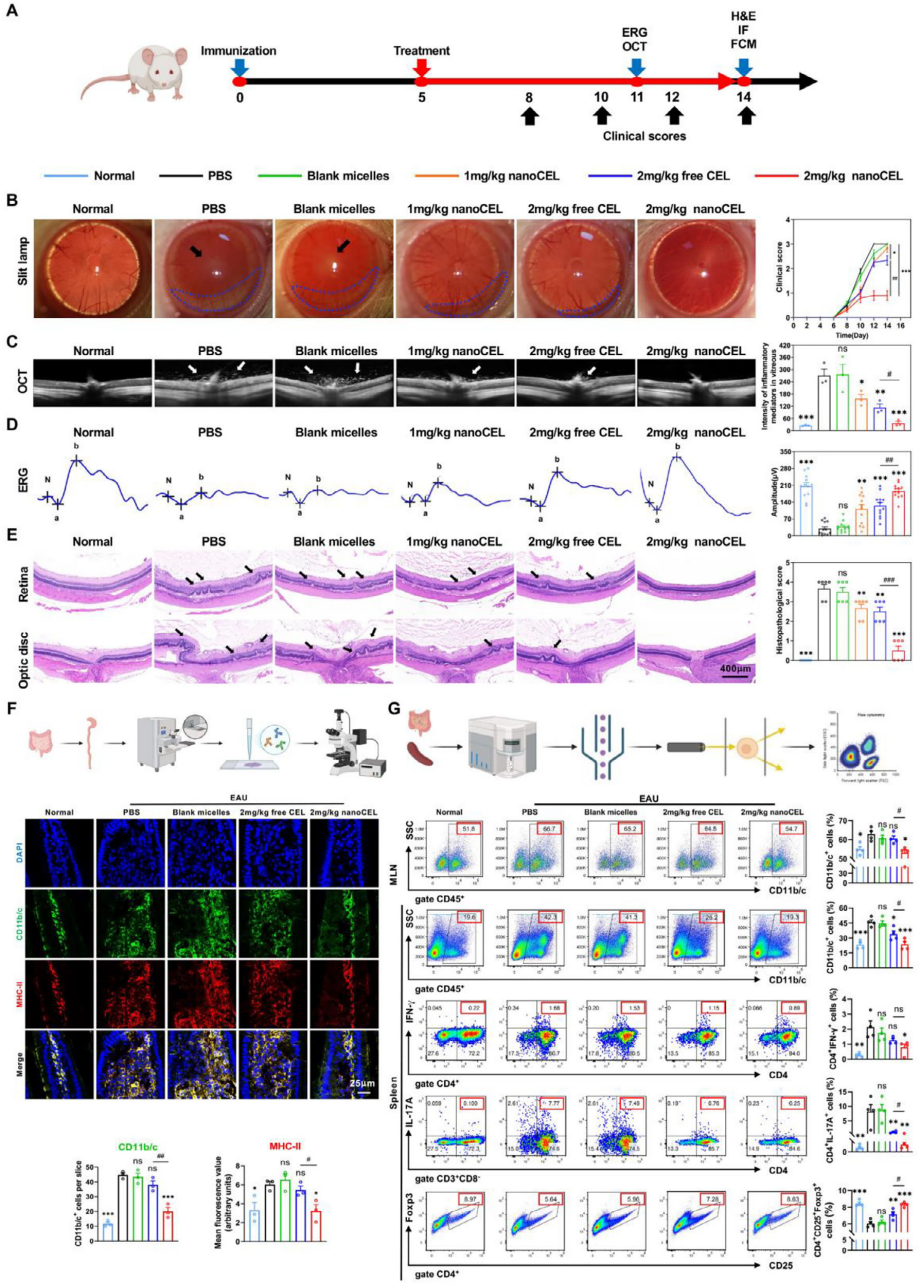
NanoCEL effectively prevents the autoimmune intraocular inflammation in an EAU rat model. (A) Schematic representation of medication and examination procedures. (B) Slit‐lamp images of EAU rats treated by various formulations at day 14 post‐immunization. The normal rats was set as a reference. Blue dotted circles and black arrows indicate the hypopyon and the pupil occlusion, respectively. Time‐course of EAU clinical scores from each group (n = 6; ^*^ *p* < 0.05; ^##^ *p* < 0.01; ^***^ *p* < 0.001). (C) Representative OCT images of EAU rats from each group at day 14 post‐immunization. The white arrow indicates the inflammatory exudate in the vitreous humor (n = 3; ns indicates no significance, ^*^ *p* < 0.05, ^**^ *p* < 0.01, ^***^ *p* < 0.001 vs. PBS group; ^#^ *p* < 0.05). (D) ERG examination of EAU rats from each group at day 14 post‐immunization. B‐wave amplitudes were analyzed at an excitation of 3 cd.s/m^2^ at day 14 post immunization (n = 6; ns indicates no significance; ^**^ *p* < 0.01, ^***^ *p* < 0.001 vs. PBS group; ^##^ *p* < 0.01). (E) Representative H&E sections of the retina from each group at day 14 post‐immunization (Black arrow indicates the retinal abnormalities, including retinal fold, infiltration of inflammatory exudate). Histopathological scores were analyzed at day 14 post‐immunization (n = 6; ns indicates no significance; ^**^ *p* < 0.01, ^***^ *p* < 0.001 vs. PBS group; ^###^ *p* < 0.001). (F) Co‐immunofluorescence staining of CD11b/c (green) and MHC‐II (red) in intestinal sections from each group at day 14 post‐immunization. The level of CD11b/c^+^ cells and MHC‐II expression of intestinal tissue from each group were analyzed (n = 3; ^*^ *p* < 0.05, ^***^ *p* < 0.001 vs. PBS group; ^##^ *p* < 0.01). (G) The proportion of CD11b/c^+^ cells (APCs) in mesenteric lymph nodes (MLN) as well as the proportion of CD11b/c^+^ cells (APCs), CD4^+^IFN‐γ^+^ T cells (Th1 cell), CD4^+^IL‐17A^+^ T cells (Th17 cell), and CD4^+^CD25^+^Foxp3^+^ T cells (Treg cell) in spleen from each group at day 14 post immunization were analyzed by flow cytometry (n = 4; ns indicates no significance; ^*^ *p* < 0.05, ^**^ *p* < 0.01, ^***^ *p* < 0.001 vs. PBS group; ^#^ *p* < 0.05).

To investigate the impacts of nanoCEL in autoimmune disorder, EAU, a well‐established autoimmune disorder model, was adopted. Following by the immunization, EAU rats were medicated with PBS, blank micelles, free CEL (2 mg/kg), nanoCEL (1 mg/kg), and nanoCEL (2 mg/kg) from day 5 to day 14 (Figure 3A). We used slit‐lamp, OCT, and ERG to assess the clinical signs and retinal changes of EAU rats. Slit‐lamp examinations showed that PBS‐treated EAU rats developed a severe intraocular inflammation accompanied with the occlusion of pupil and hypopyon (Figure 3B). The treatment of blank micelles failed to attenuate the intraocular inflammation of EAU rats. Free CEL and nanoCEL dose‐dependently ameliorated the intraocular inflammation of EAU rats. Notably, the treatment of nanoCEL (2 mg/kg) exhibited the superior therapeutic efficacy in terms of the lowest clinical score among all groups. Compared to other groups, almost no hypopyon and anterior inflammatory responses were observed in the nanoCEL group (2 mg/kg). Similarly, OCT observation indicated that there were numerous inflammatory exudations in the vitreous humor and retina after the treatment of PBS and blank micelles. Treatments of free CEL (2 mg/kg) and nanoCEL (1 mg/kg and 2 mg/kg) markedly reduced the production of inflammatory exudations. Moreover, the treatment of nanoCEL at the concentration of 2 mg/kg yielded the lowest inflammatory exudations in the vitreous humor and retina as compared to other groups (Figure 3C). Besides, ERG analysis confirmed that the treatment of nanoCEL at the concentration of 2 mg/kg provided better vision during the progression of EAU than other groups (Figure 3D). Consistently, lower levels of histopathological features in terms of inflammatory cell infiltration and retinal fold corroborated with the ameliorated uveitis in 2 mg/kg of nanoCEL‐treated rats (Figure 3E). Compared with other groups, the 2 mg/kg of nanoCEL‐treated group developed less EAU in terms of histological score.

To determine whether nanoCEL targeted APCs to ameliorate EAU, we medicated nanoCEL at day 8 post‐immunization (Figure S2A). Interestingly, unlike the early phase of medication at day 5, 2 mg/kg of free CEL treatment failed to attenuate clinical signs of EAU, and the severity of EAU was not significantly different from that in the PBS group (Figure S2B). Additionally, 2 mg/kg of nanoCEL treatment partially suppressed the severity of EAU, corresponding to the lower histopathological scores (Figure S2C). Based on these data, it is reasonable to believe that the suppression of EAU by nanoCEL treatment is in a time‐dependent manner. Since the primary function of APCs is to initiate and amplify EAU, APCs targeting by nanoCEL during the early phase of disease development (day 5) significantly suppressed the severity of EAU, but exhibited less therapeutic efficacy when medicated at the later phase of disease development (day 8). This result suggested that the function of peripheral macrophages and T cells might contribute to the sustainable intraocular inflammation once APCs were activated at the onset of EAU. We thereafter assessed the populations of APCs and the expression of MHC‐II in intestinal tissue. The absolute numbers and populations of CD11b/c^+^ APCs were greatly elevated in PBS‐treated EAU rats compared with normal rats, as evidenced by immunofluorescence and flow cytometry assay. Notably, 2 mg/kg of nanoCEL treatment severely reduced the numbers and populations of CD11b/c^+^ APCs with a parallel of the reduced level of MHC‐II expression (Figure 3F). Thus, oral administration of nanoCEL rather than free CEL at the dosage of 2 mg/kg exhibited the robust inhibitory effect of antigen‐presenting capacity.

### 3.6 NanoCEL Prevents Pathogenic T Cell Differentiation

Given that EAU has been regarded as a Th1‐ and Th17‐mediated autoimmune disorder, we analyzed the T cell subset in the spleen and cervical lymph nodes (CLN) from each group. In agreement with the previous study, PBS‐treated EAU rats markedly increased the frequency of CD11b/c^+^ APCs, CD4^+^INF‐γ^+^ cells (Th1), and CD4^+^IL17A^+^ (Th17) cells, while reduced the frequency of CD4^+^CD25^+^Foxp3^+^ regulatory T cells (Treg) in spleen and CLN as compared to normal rats (Figure 3G; Figure S3). Consistent with the reduced frequency of APCs in intestinal tissue and mesenteric lymph nodes (MLN), 2 mg/kg of nanoCEL treatment also significantly reduced the frequency of CD11b/c^+^ APCs in spleen and CLN. Additionally, 2 mg/kg of nanoCEL treatment exhibited a more profound inhibitory effect of pathogenic Th1 and Th17 cells in spleen and CLN with respect to free CEL treatment. By contrast, the frequency of Treg cells was greatly elevated in the spleen and CLN with 2 mg/kg of nanoCEL treatment. Together, all these results indicated that oral administration of nanoCEL effectively impaired the pathogenic T cell differentiation (Th1 and Th17), consequently attenuating the intraocular inflammation.

### 3.7 NanoCEL Protects the Integrity of the Blood‐Retinal Barrier (BRB) and RPE Tight Junction in EAU Rats

**FIGURE 4:**
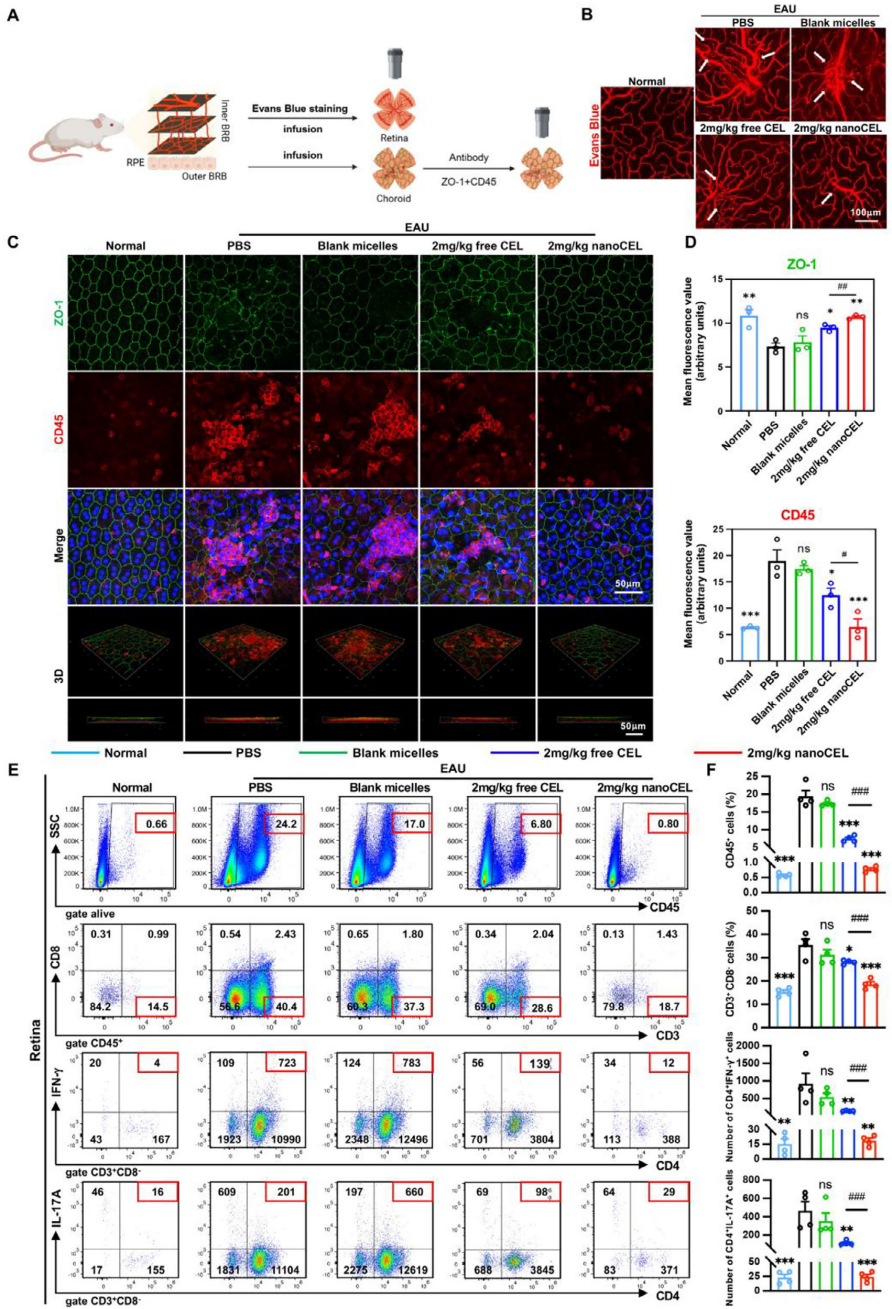
NanoCEL protects the integrity of the blood‐retinal barrier (BRB) and RPE tight junction in EAU rats. (A) Schematic diagram of the detection of blood‐retinal barrier (BRB) integrity. (B) Representative images of retina flat mounts after perfusion with Evans blue at day 14 post‐immunization from the normal rats and EAU rats treated by various formulations. (C) ZO‐1 (green) and CD45 (red) co‐immunofluorescence staining of RPE flat mounts from each group. (D) Quantitative analysis of ZO‐1 and CD45 from each group (n = 3; ns indicates no significance; ^*^ *p* < 0.05, ^**^ *p* < 0.01, ^***^ *p* < 0.001 vs. PBS group; ^#^ *p* < 0.05, ^##^ *p* < 0.01); (E) The proportion of CD45^+^ cells (leukocytes), CD3^+^CD8^−^ T cells (CD4^+^ T cells), CD4^+^IFN‐γ^+^ T cells (Th1 cell), and CD4^+^IL‐17A^+^ T cells (Th17 cell) in retina from each group at day 14 post‐immunization were analyzed by flow cytometry (n = 4; ns indicates no significance; ^*^ *p* < 0.05, ^**^ *p* < 0.01, ^***^ *p* < 0.001 vs. PBS group; ^###^ *p* < 0.001).

To visualize the leakage of BRB in rats, the Evans blue dye was injected intravenously via the tail vein (Figure 4A). As shown in Figure 4B, there was almost no dye leakage from retina in normal rats, while apparent dye leakage was observed in the PBS‐treated EAU rats, indicating that the disruption of BRB acted a vital role in such leakage. Blank micelles treatment failed to reduce the dye leakage from retina, and 2 mg/kg of free CEL and 2 mg/kg of nanoCEL treatment both resulted in a significant reduction of dye leakage from retina. Of note, nanoCEL treatment had the strongest protective effect of BRB with minimal dye leakage. The disruption of BRB, especially outer BRB, might be ascribed to the disorganization and destroy of RPE tight junction proteins. Comparing with normal rat, the expression of ZO‐1 was disrupted and decreased obviously in the RPE layer, accompanied with numerous CD45^+^ leukocyte infiltrations in PBS‐treated EAU rat (Figure 4C,D). Similarly, blank micelles‐treated EAU rat displayed a severe destroy of outer BRB and CD45^+^ leukocyte infiltration in the RPE layer. The treatment of free CEL slightly rescued the BRB with few CD45^+^ leukocyte infiltrations in the RPE layer. In contrast, nanoCEL treatment almost maintained the integrity of BRB with no CD45^+^ leukocyte breakthrough in the RPE layer. Based on these data, it is reasonable to believe that oral administration of nanoCEL effectively protects the integrity of BRB and RPE tight junction in EAU rats.

### 3.8 NanoCEL Blockades the Infiltration of Peripheral Immune Cells in Retina and Curtails the Activation of Retinal Glia Cells

In light of the effectiveness of nanoCEL in the maintenance of the BRB integrity in the EAU rat, we then investigated the impact of nanoCEL on the infiltration of peripheral immune cells in the retina. There were few CD45^+^, CD4^+^, CD4^+^IFN‐γ^+^ (Th1) and CD4^+^IL‐17A^+^ (Th17) cells in retina from normal rats. Owing to the disruption of BRB, the numbers and frequencies of CD45^+^, CD4^+^, CD4^+^IFN‐γ^+^ (Th1) and CD4^+^IL‐17A^+^ (Th17) cells in retina increased markedly in PBS‐treated EAU rats (Figure 4E). Both 2 mg/kg of free CEL treatment and 2 mg/kg of nanoCEL treatment effectively reduced the infiltration of these peripheral immune cells in the retina, while nanoCEL treatment exhibited a more potent inhibitory ability of infiltration of these peripheral immune cells in the retina than free CEL treatment. Immunofluorescence co‐staining of CD4 and IL‐17 also confirmed that there was almost invisible infiltration of Th17 cells in the retina from nanoCEL‐treated EAU rat (Figure S4A). Apart from the infiltration of peripheral immune cells in the retina, the resident retinal glia cells, including microglia and astrocytes, were abnormally activated in the EAU rat. Accordingly, nanoCEL treatment significantly suppressed the activation of microglia and astrocytes, as indicated by the low expression of Iba‐1 and GFAP (Figure S4B,C). All these data indicated that oral administration of nanoCEL (2 mg/kg) remarkably reduced the infiltration of peripheral immune cells in the retina and curtailed the activation of retinal glia cells in EAU rats.

### 3.9 NanoCEL Enhances Oral Bioavailability and Alters Drug Biodistribution

**FIGURE 5:**
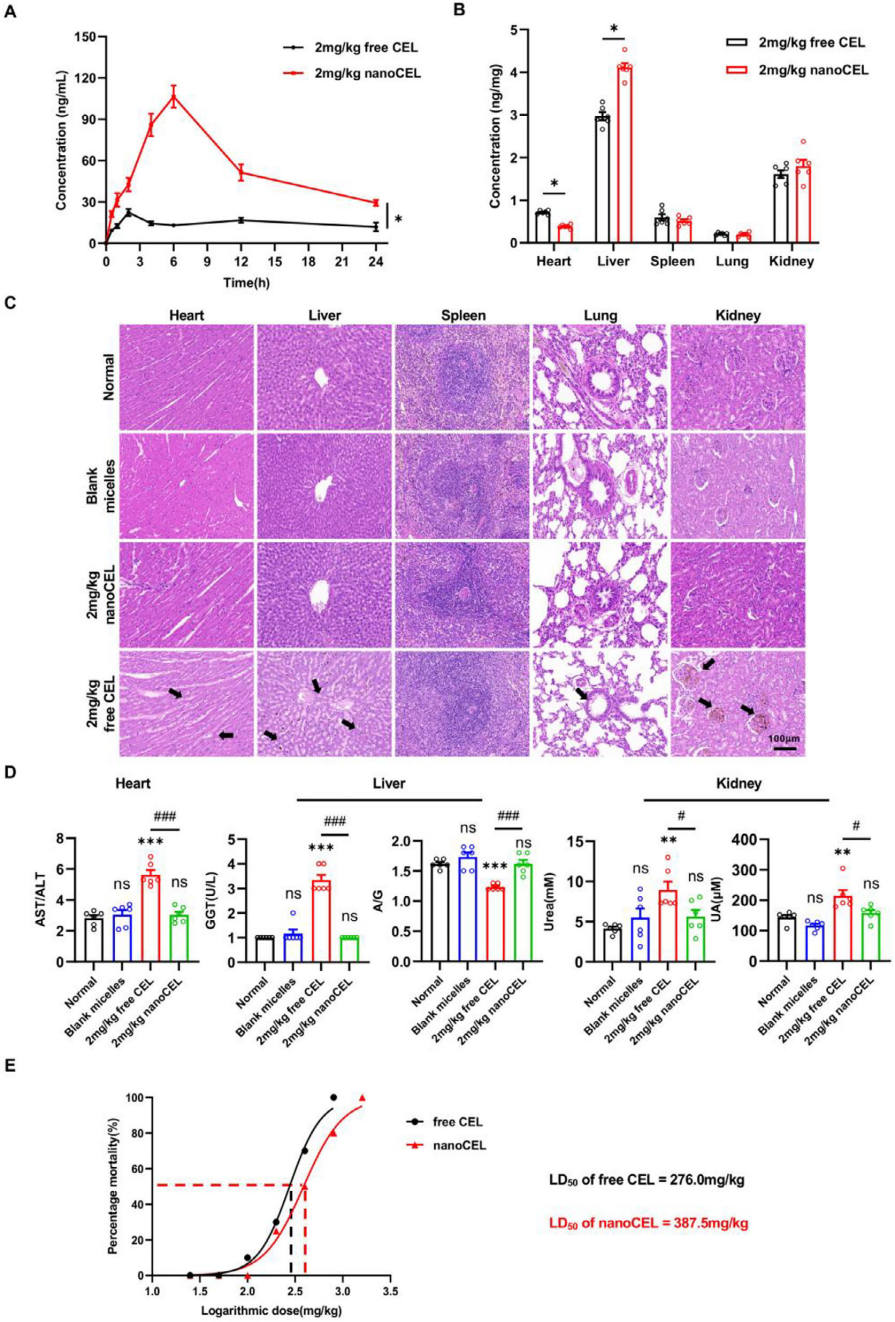
NanoCEL improves the oral bioavailability and biocompatibility compared to CEL. (A) Plasma drug concentrations over a 24 h period after oral administration of 2 mg/kg free CEL or 2 mg/kg nanoCEL (n = 6; ^*^ *p*< 0.05). (B) Drug concentration in major organs over a 6 h period after oral administration of 2 mg/kg free CEL or 2 mg/kg nanoCEL (n = 6; ^*^ *p* < 0.05). (C) H&E sections of heart, liver, spleen, lung, and kidney at day 14 post oral administration of PBS, blank micelles, 2 mg/kg nanoCEL, and 2 mg/kg free CEL. The arrow indicates myocardial cell atrophy and myofibrillar loss in heart tissue; atrophy of hepatic cells and blood sinus dilatation in liver tissue; and glomerular atrophy in kidney tissue. (D) Serum levels of aspartate aminotransferase/alanine transaminase (AST/ALT), gamma‐glutamyl transferase (GGT), albumin to globylin ratio (A/G), urea, and uric acid (UA) at day 14 post oral administration of PBS, blank micelles, 2 mg/kg nanoCEL, and 2 mg/kg free CEL. (n = 6; ns indicates no significance; ^**^ *p* < 0.01, ^***^ *p* < 0.001 vs. Normal group; ^#^ *p* < 0.05, ^###^ *p* < 0.001). (E) The LD_50_ value of oral administration of free CEL and nanoCEL.

We thereafter measured the pharmacokinetics and drug biodistribution after oral administration of free CEL and nanoCEL. As presented in Figure 5A, after oral administration of free CEL (2 mg/kg) and nanoCEL (2 mg/kg), the plasma drug concentration increased rapidly and peaked at 2 and 6 h, respectively, and gradually decreased in the following 24 h. Following by the analysis of mean drug concentration‐time curves, we calculated the pharmacokinetic parameters for free CEL and nanoCEL (Table 1). In contrast to free CEL, nanoCEL exhibited a significantly higher value of T_max_, C_max,_ and AUC, corresponding to the enhanced drug bioavailability. Interestingly, oral administration of nanoCEL exhibited a distinct tissue distribution manner with respect to that of free CEL (Figure 5B). As compared to free CEL, nanoCEL significantly decreased the drug level in the heart but greatly increased the drug accumulation in the liver. Despite the relatively higher drug concentration in the liver for nanoCEL, it seems to suggest that nanoCEL was dominantly uptaken by liver macrophages but not hepatocytes, as evidenced by a significantly higher cellular uptake of nanoCEL in RAW264.7 macrophages than that in BRL‐3A cells (Figure S5). Conversely, free CEL exhibited higher cellular uptake in BRL‐3A cells than in RAW264.7 macrophages, which possibly caused potential liver toxicity. Thus, oral administration of nanoCEL remarkably enhanced drug bioavailability and altered the drug tissue biodistribution with respect to free CEL.

**TABLE 1:** Pharmacokinetic parameters of nanoCEL in comparison with free CEL (2 mg/kg). Data were presented as mean ± SEM and analyzed using a two‐tailed Student's t‐test (n = 6; ^**^ *p* < 0.01, ^***^ *p* < 0.001).

Parameters	2 mg/kg Free CEL	2 mg/kg NanoCEL
T_max_(h)	2.20 ± 1.10	5.33 ± 1.03***
C_max_(ng/mL)	23.42 ± 6.22	110.08 ± 15.81***
MRT(h)	11.91 ± 1.01	9.86 ± 0.21**
AUC(h*ng/mL)	330.17 ± 111.25	1333.41 ± 167.21***

### 3.10 NanoCEL Reduces the Systemic Drug Toxicity and Improves Biosafety

We next assessed the systemic toxicity and biosafety of free CEL or nano CEL in rats via an oral administration route. As expected, animals exposed to blank micelles displayed no visible histological changes in heart, liver, spleen, lung, and kidney (Figure 5C). Also, blank micelles treatment did not significantly alter the levels of AST/ALT, GGT, A/G, Urea, and UA as compared to normal rats (Figure 5D). Notably, 2 mg/kg of free CEL treatment induced myocardial cell atrophy accompanied with the significantly elevated levels of AST/ALT, indicating the obvious cardiotoxicity. Also, 2 mg/kg of free CEL‐treated rats exhibited the diffused edema and hepatocyte necrosis in the liver aligned with elevated GGT levels and decreased A/G levels, implying notable hepatotoxicity. Additionally, there was apparent injury to glomeruli with raised levels of urea and UA in 2 mg/kg of free CEL‐treated rats, suggesting the robust nephrotoxicity. By contrast, 2 mg/kg of nanoCEL‐treated rats showed no visible signs of cardiotoxicity, hepatotoxicity, and nephrotoxicity. Furthermore, the LD_50_ value of rats significantly increased from 276 mg/kg for free CEL to 387.5 mg/kg for nanoCEL, suggesting the great tolerance (Figure 5E). Collectively, the encapsulation of CEL into micelles greatly reduced the toxicity of CEL to major organs and provided great biosafety.

## 4 Discussion

APCs‐targeted drug delivery is a powerful immunotherapy for autoimmune disorders that has shown promise in early clinical trials [56]. By contrast with conventional immunosuppressive therapies, elicited tolerogenic APCs by nanomedicine may be capable of restraining the cytotoxic and inflammatory responses in an antigen‐specific manner, thus providing a precise strategy to treat various autoimmune disorders. However, several physicochemical parameters of nanomedicine, including size, shape, charge, and so on, significantly influence the ability to boost APCs targeting and response [57, 58, 59]. In this study, we fabricated and optimized a nanomedicine, namely nanoCEL, to achieve APCs‐targeted delivery, subsequently modulating the T cell differentiation to alleviate autoimmune uveitis. The optimized nanoCEL had the mean diameter of 37.06 ± 0.12 nm and the slight negative charge of −1.83 ± 0.24 mV. The drug payload of nanoCEL was over 5%, and nanoCEL displayed a typical pH‐responsive drug release behavior. The utilization of micelles to manipulate APCs exhibited several advantages as follows: 1) achieving the relatively high drug payload, 2) precise control release of drug from nanomedicine, and 3) reduction of off‐target effects [60, 61, 62].

Efficient cellular uptake of nanomedicine by APCs is prerequisite to modulating the subsequent immune response. We validated that nanoCEL remarkably enhanced cellular uptake of APCs and exhibited a more profound inhibitory effect of antigen‐presenting ability in APCs with respect to free CEL. Previous studies have been documented that nanoparticles with diameter less than 100 nm can be efficiently internalized into APCs by clathrin‐mediated endocytosis, thus modulating the immune response [63, 64]. Following by oral administration, the proposed nanoCEL efficiently penetrated the mucous epithelial barrier and targeted APCs in the intestine. PEGylation of nanoparticles enabled the efficient mucus penetration owing to the possibly reduced interaction between the nanoparticle and mucus. A recent study also verified that negatively charged micelles exhibited superior efficacy in targeting the lymphatics following subcutaneous administration [65].

Given the outperformance of nanoCEL targeting APCs, we showed that the intraocular inflammation in the EAU rat was significantly attenuated by nanoCEL treatment at the early phase (day 5). We showed that the expression of MHC‐II and the number of CD11b/c^+^ APCs were highly increased in the intestine and MLN of EAU rats, which was consistent with in vitro results. Interestingly, nanoCEL treatment at later phase (day 8) of disease development significantly compromised its therapeutic efficacy. Pilot studies reported that the depletion of local APCs in the retina (i.e., microglia) at the early stage of EAU remarkably reduced leukocyte infiltration into the retina, while the depletion of microglia had no effect on the severity and duration of EAU after intraocular inflammation had already happened [66]. This indicated that APCs were the initiator of EAU onset, and the pathogenic T cells, as well as other immune cells involved of the amplification of inflammatory response. We also demonstrated that nanoCEL treatment protected the integrity of blood‐retinal barrier (BRB) and RPE tight junction in EAU rats. Under physiological conditions, the BRB protected the retina from plasma proteins as a result of the immune privilege. The activated pathogenic T cells in the circulation of EAU rats recruited other leukocytes that induced a persistent and marked breakdown of the BRB [67, 68]. Our data showed that nanoCEL treatment significantly decreased the population of pathogenic T cells in the spleen, CLN, and retina. To validate the possible therapeutic mechanism of nanoCEL via gut‐retina axis, oral administration of nanoDIR showed that the fluorescent signal was primarily located at the intestine, mesenteric lymph node (MLN), liver, and kidney but not in the eyeball at 8 h (Figure S6). This result seems to suggest that oral administration of nanoCEL did not traffic to eyeball directly. Taking into consideration of the specific accumulation of nanoCEL at mesenteric lymph nodes (MLN) and robust suppression of antigen‐presenting ability in intestinal APCs after oral administration, we thereafter analyzed the subtype cells alteration of peripheral blood mononuclear cell (PBMC) (Figure S7). Interestingly, the proportion of Th1, Th17, and B cells in the blood decreased remarkably in the nanoCEL group as comparison with EAU rat. This result strongly indicated that the proposed nanoCEL might exert this therapeutic efficacy via an MLN‐blood‐retina pathway. Meanwhile, pilot studies have been documented that the dysfunction of gut microbiota plays a critical role in the pathogenesis of EAU, and the modulation of gut microbiota is a promising strategy for autoimmune uveitis therapy [69, 70]. In this study, oral administration of nanoCEL might be able to alter the gut microbiota map, and thus contributing its therapeutic efficacy. Thus, the exact mechanism of nanoCEL via oral administration route needed to be elucidated in future work. In addition, oral administration of nanoCEL greatly enhanced drug bioavailability and altered the biodistribution of CEL, thus minimizing CEL‐related off‐target toxicity and improving biosafety.

## 5 Conclusion

In conclusion, we engineered an oral intestinal APCs‐targeted nanodrug delivery system by the straightforward encapsulation of CEL into polymeric micelles, termed nanoCEL, for alleviating autoimmune uveitis. Such nanoCEL easily penetrated the mucus barrier and targeted APCs in the intestine after oral administration. The treatment of nanoCEL (2 mg/kg) overwhelmingly ameliorated autoimmune intraocular inflammation in EAU rats by significantly reducing the antigen‐presenting capacity of APCs and impairing pathogenic T cell differentiation. Also, nanoCEL treatment protected the integrity of BRB and RPE tight junctions in EAU rats, thereby reducing the infiltration of peripheral immune cells into the retina and suppressing the activation of retinal glia cells, including microglia and astrocytes. In contrast to free CEL, oral administration of nanoCEL greatly enhanced drug bioavailability and altered drug biodistribution, thus minimizing systemic toxicity and improving biosafety. This might be a promising strategy for treating AU in the clinic.

## Conflicts of Interest

The authors declare no conflict of interest.

## Supporting information




**Supporting File**: advs74163‐sup‐0001‐SuppMat.docx.

## Data Availability

The data that support the findings of this study are available from the corresponding author upon reasonable request.
