# Supplementary material for: Oral Celastrol Nanomedicine Targeting Intestinal Antigen‐Presenting Cells to Effectively Mitigate Autoimmune Uveitis via Gut‐Retina Axis
Source: Adv Sci (Weinh). 2026 Feb 3;13(20):e19503. doi: 10.1002/advs.202519503 (PMC13067824; doi:10.1002/advs.202519503)
Supplement: Supplementary file 1 — Supporting File: advs74163‐sup‐0001‐SuppMat.docx. [file ADVS-13-e19503-s001.docx]

**Supporting information**

**Oral celastrol nanomedicine targeting intestinal antigen-presenting cells to effectively mitigate autoimmune uveitis via gut-retina axis**

Jinrun Chen^1^, Yuqin Wu^1^, Bofei Xu^1^, Jiabei Hou^1^, Yijing Li^1^, Yuhan Hu^2^, Yutuo Zhu^1^, Wenqiao Zhang^1^, Shuqi Feng^1^, Huanting Jin^1^, Yuchen Cheng^1^, Yuanyuan Jin^1^, Jianhong Zhou^1*^, Xingyi Li^1*^

1. National Engineering Research Center of Ophthalmology and Optometry, Eye Hospital, Wenzhou Medical University, Wenzhou, 325027, China
2. State Key Laboratory of Medicinal Chemical Biology, Key Laboratory of Bioactive Materials, Ministry of Education and College of Life Sciences, Nankai University, Tianjin 300071, China.

^*^ Correspondence to: lixingyi_1984@mail.eye.ac.cn (X. Li), zjh23er@eye.ac.cn (J. Zhou).

**
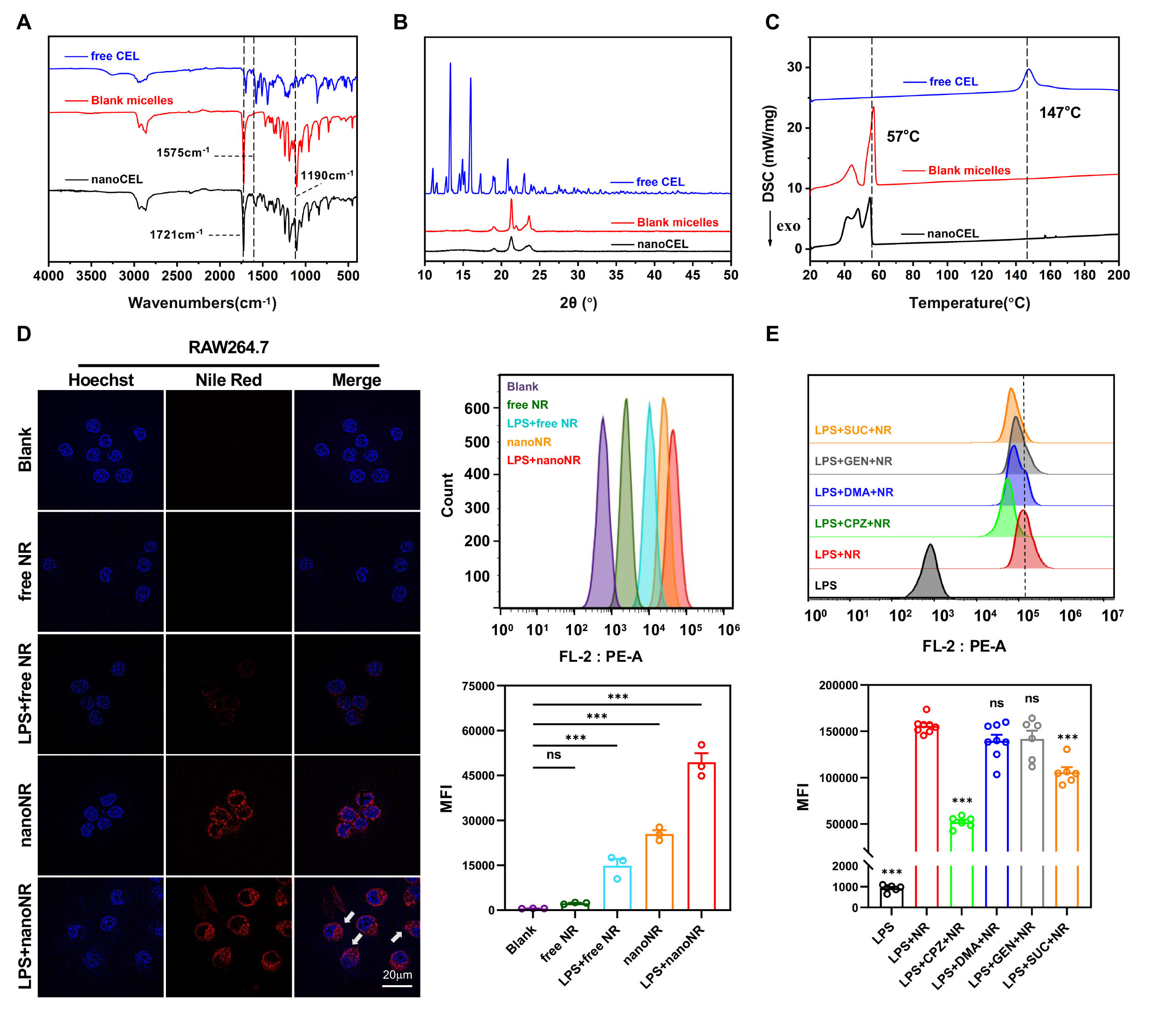
**

**Figure S1. (A)** FTIR spectra of free CEL, blank micelles and nanoCEL. **(B)** XRD spectra of free CEL, blank micelles and nanoCEL. **(C)** DSC profiles of free CEL, blank micelles and nanoCEL. **(D)** CLMS image and flow cytometry analysis of cellular uptake in RAW264.7 macrophages at 15 min. Nile Red (NR) fluorescence is depicted in red. (n = 6; ns indicates no significance; ^***^p < 0.001 *vs.* Blank group). **(E)** Flow cytometry analysis of cellular uptake in RAW264.7 macrophages with treatment of 10 μg/mL Chlorpromazine (CPZ), 10 μM 5-(N, N-dimethyl) amiloride (DMA), 50 μg/mL genistein (GEN) or 154 mg/mL sucrose (SUC) (n = 6; ns indicates no significance; ^***^p < 0.001 *vs.* LPS+NR group).


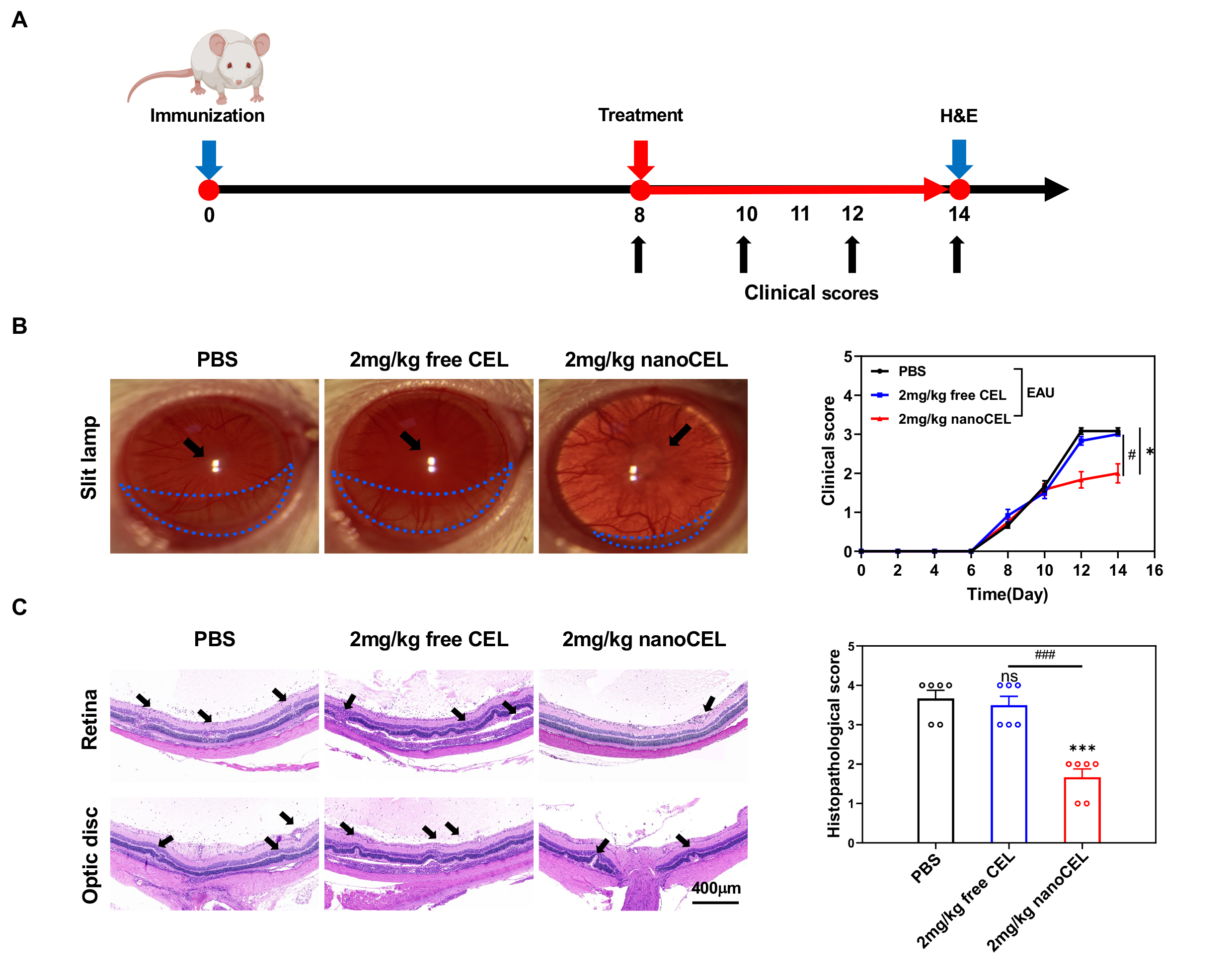


**Figure S2. (A)** Schematic representation of medication and examination. **(B)** Slit lamp image of EAU rats treated by PBS, 2 mg/kg free CEL and 2 mg/kg nanoCEL at day 14 post-immunization. Blue dotted circle and black arrows indicate the hypopyon and the occlusion of the pupil, respectively. Time-course of EAU clinical scores from each group (n = 6; ^*^p < 0.05; ^#^p < 0.05). **(C)** Representative H&E sections of the retina from each group at day 14 post-immunization (Black arrows indicate the retinal abnormalities including retinal fold, infiltration of inflammatory exudate). Histopathological scores were analyzed at day 14 post-immunization (n = 6; ns indicates no significance; ^**^p < 0.01, ^***^p < 0.001 *vs.* PBS group; ^###^p < 0.001).


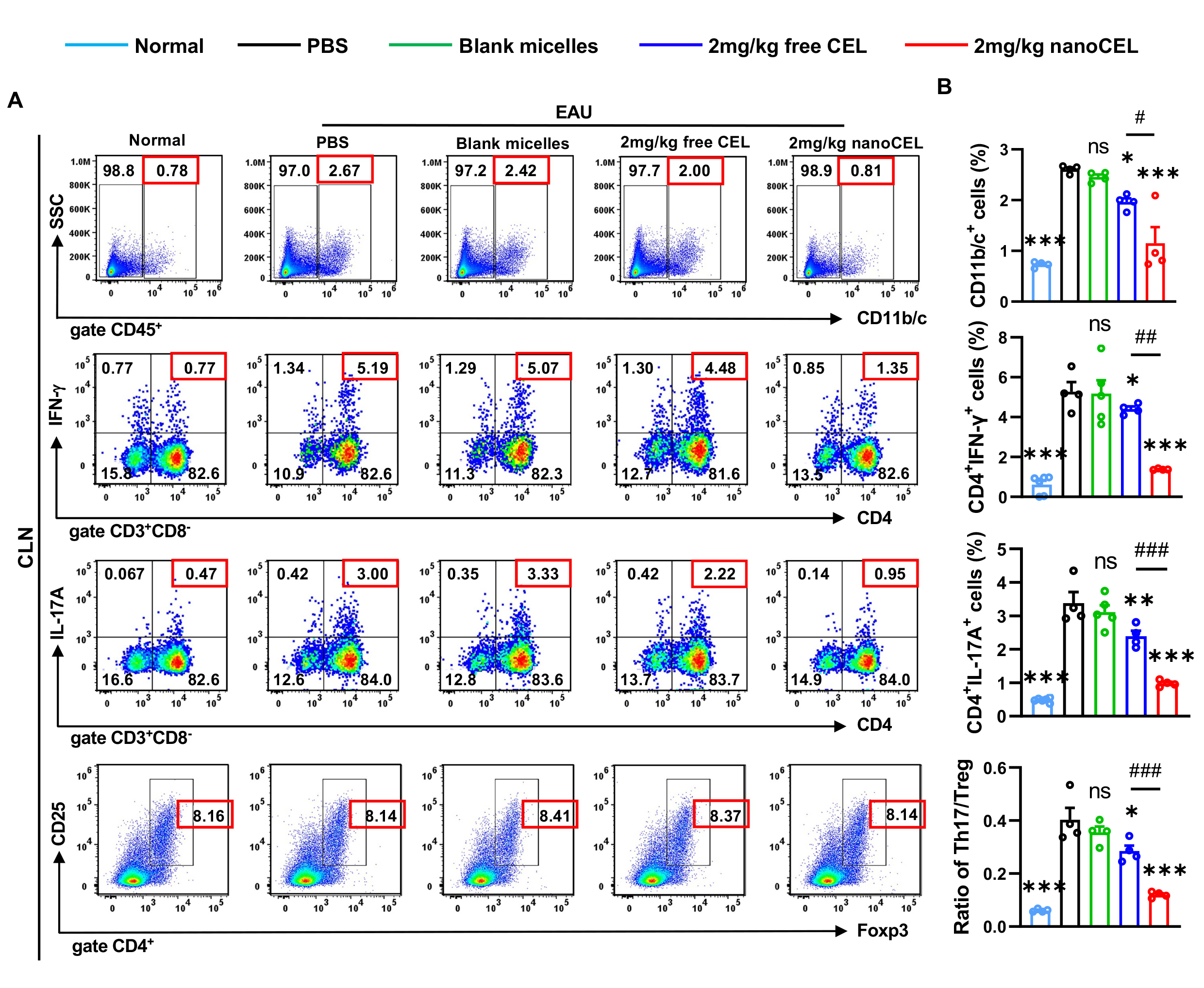


**Figure S3.** The proportion of CD11b/c^+^ cells (APCs), CD4^+^IFN-γ^+^ T cells (Th1 cell), CD4^+^IL-17A^+^ T cells (Th17 cell), and CD4^+^CD25^+^Foxp3^+^ T cells (Treg cell) in cervical lymph nodes (CLN) from each group at day 14 post-immunization were analyzed by flow cytometry (n = 4; ns indicates no significance; ^*^p < 0.05, ^**^p < 0.01, ^***^p < 0.001 *vs.* PBS group; ^#^p < 0.05, ^##^p < 0.01, ^###^p < 0.001).


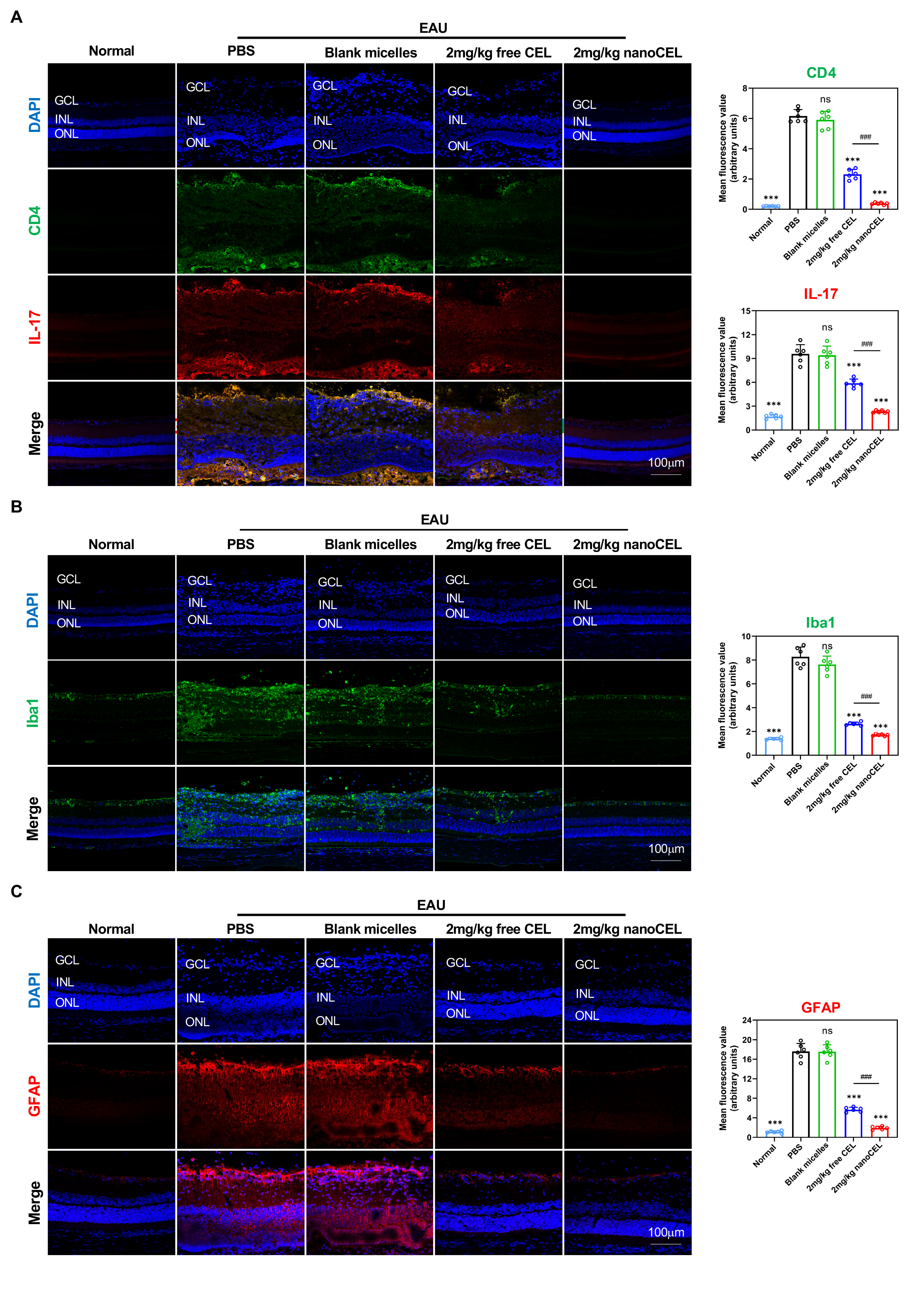


**Figure S4.** **(A)** CD4 (green) and IL-17 (red) co-immunofluorescence staining of retinal sections at day 14 post-immunization from each group. (n = 3; ns indicates no significance; ^***^p < 0.001 *vs.* PBS group; ^###^p < 0.001). **(B)** Iba1 (green) and **(C)** GFAP (red) expression in retinal tissues in each group. (n = 3; ns indicates no significance; ^***^p < 0.001 *vs.* PBS group; ^###^p < 0.001)


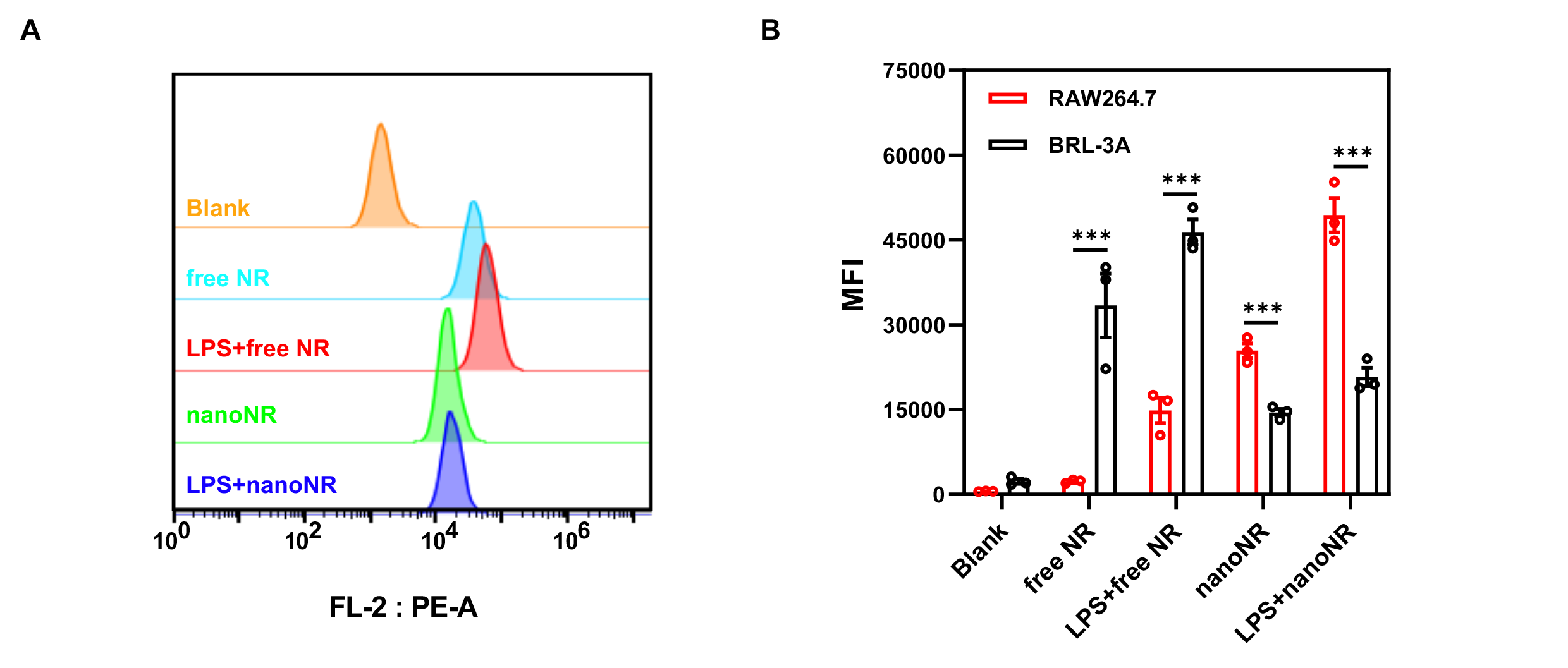


**Figure S5.** **(A)** Flow cytometry analysis of cellular uptake in BRL-3A cells (rat hepatocyte). **(B)** Quantitative analysis of cellular uptake in RAW264.7 macrophages and BRL-3A cells (n = 3; ^***^p < 0.001)


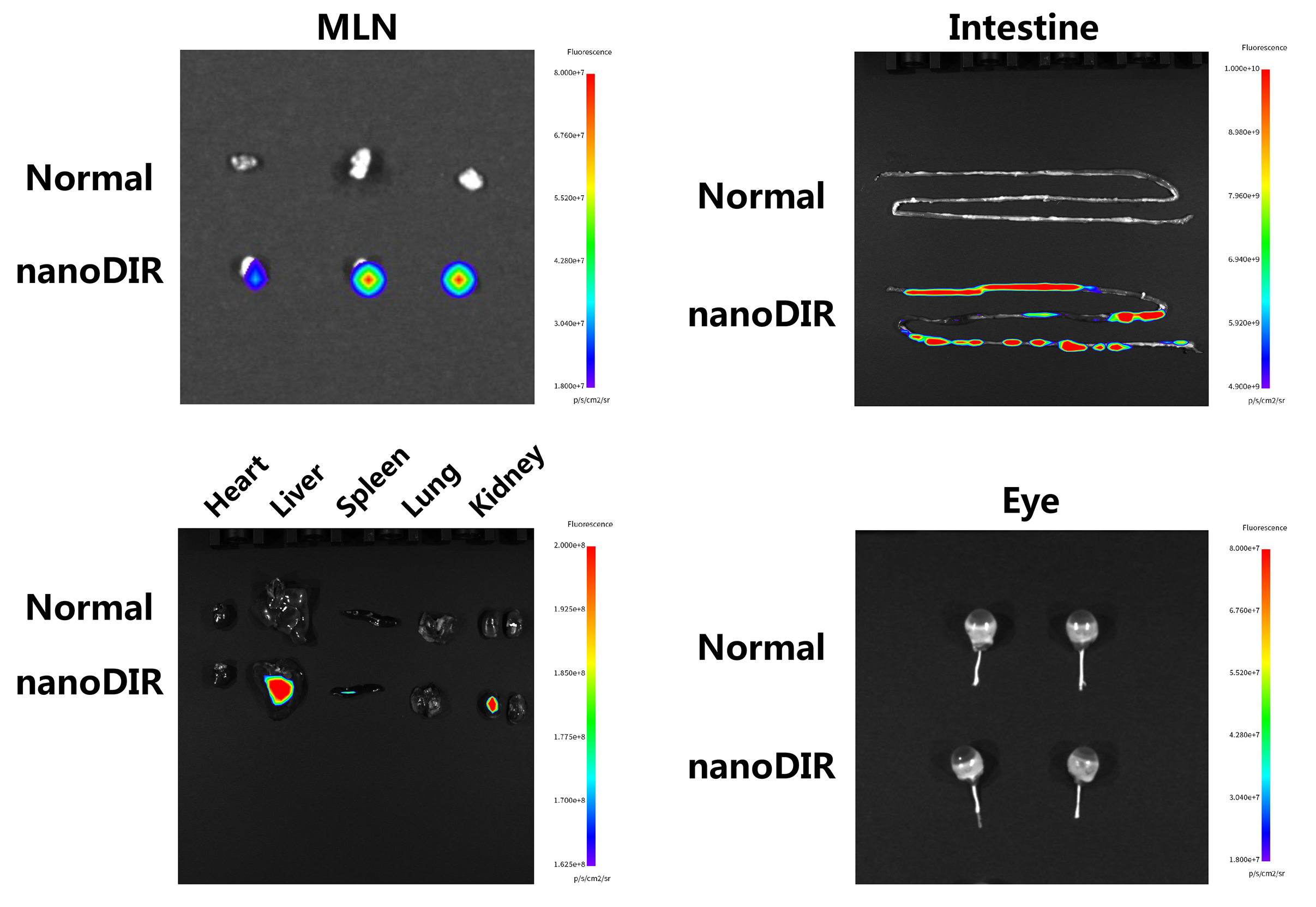


**Fig.S6** *In vivo* distribution of nanoDIR at different organs via oral administration at 8h


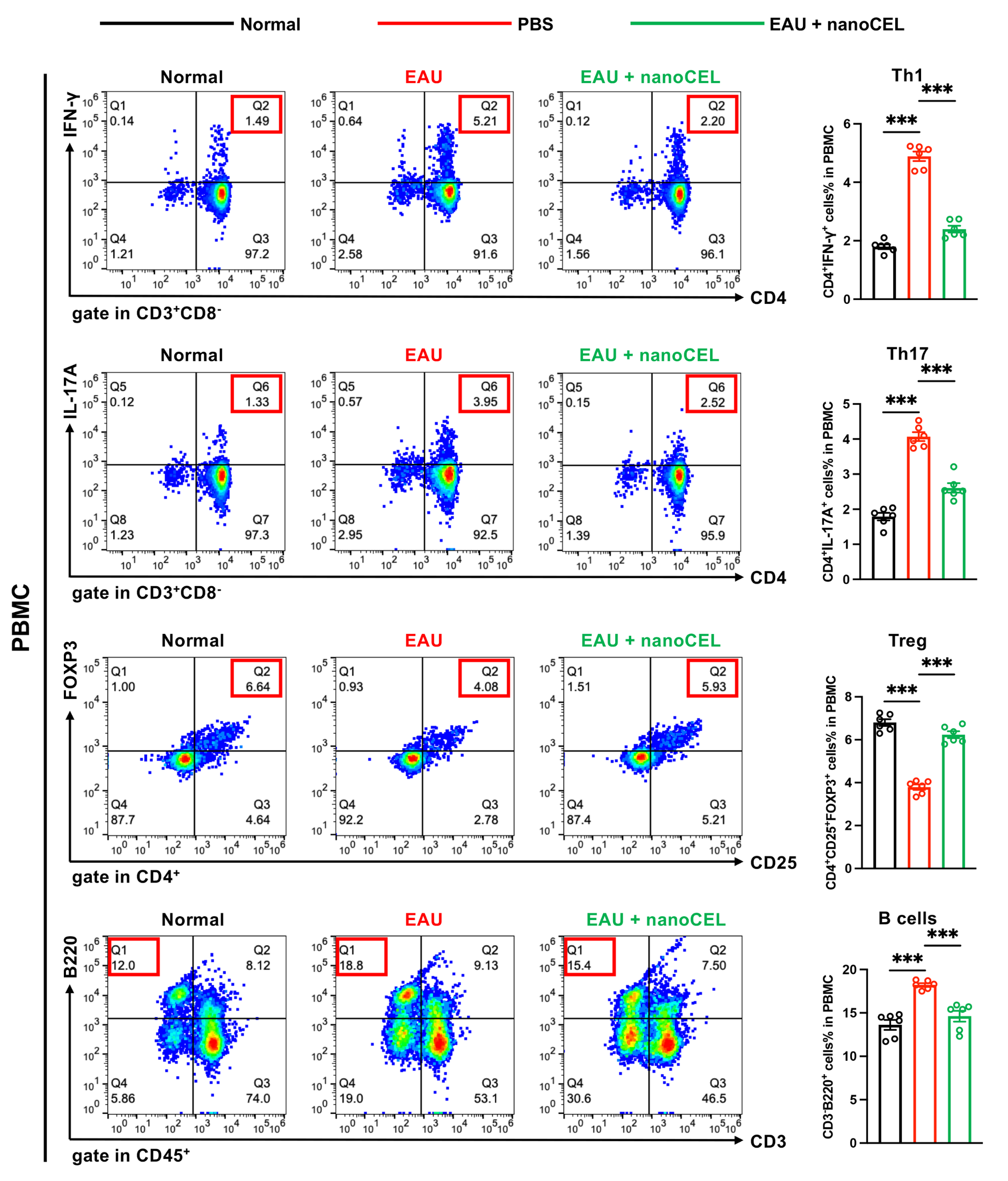


**Figure S7.** The proportion of CD4^+^IFN-γ^+^ T cells (Th1 cell), CD4^+^IL-17A^+^ T cells (Th17 cell), CD4^+^CD25^+^Foxp3^+^ T cells (Treg cell) and CD45^+^CD3^-^B220^+^ B cells (B cells) in peripheral blood mononuclear cell (PBMC) from each group at day 12 post-immunization were analyzed by flow cytometry (n = 6; ^***^p < 0.001).

**Table S1.** Effect of drug/polymer feed ratio on the parameters of nanoCEL (n = 3)

| Drug/polymer feed ratio | Mean particle size | Zeta potential (mv) | EE (%) | LC(%) |
| --- | --- | --- | --- | --- |
| 1/1  1/2  1/3  1/5  1/10  1/20 | 134.40 ± 1.56  119.60 ±0.15  51.96 ± 0.31  40.29 ± 0.17  37.06 ± 0.12  34.33 ± 0.18 | -11.90 ± 0.40  -10.90 ± 0.27  -5.88 ± 0.11  -3.14 ± 0.30  -1.83 ± 0.24  -1.55 ± 0.10 | 13.10 ± 1.00  17.65 ± 1.97  43.77 ± 0.79  82.61 ± 1.36  98.66 ± 4.68  89.54 ± 7.94 | 11.58 ± 0.78  8.10 ± 0.83  12.73 ± 0.20  14.18 ± 0.20  8.98 ± 0.39  4.28 ± 0.36 |
